# Characteristics and one year outcomes of melioidosis patients in Northeastern Thailand: a prospective, multicenter cohort study

**DOI:** 10.1016/j.lansea.2022.100118

**Published:** 2022-11-25

**Authors:** Narisara Chantratita, Rungnapa Phunpang, Atchara Yarasai, Adul Dulsuk, Thatcha Yimthin, Lauren A. Onofrey, Taylor D. Coston, Ekkachai Thiansukhon, Seksan Chaisuksant, Kittisak Tanwisaid, Somchai Chuananont, Chumpol Morakot, Narongchai Sangsa, Sunee Chayangsu, Wirayut Silakun, Noppol Buasi, Ploenchan Chetchotisakd, Nicholas P.J. Day, Ganjana Lertmemongkolchai, T. Eoin West

**Affiliations:** a Department of Microbiology and Immunology, Faculty of Tropical Medicine, Mahidol University, Bangkok, Thailand; b Mahidol-Oxford Tropical Medicine Research Unit, Faculty of Tropical Medicine, Mahidol University, Bangkok, Thailand; c Division of Pulmonary, Critical Care & Sleep Medicine, Department of Medicine, University of Washington, Seattle, WA, USA; d Department of Medicine, Udon Thani Hospital, Udon Thani, Thailand; e Department of Medicine, Khon Kaen Regional Hospital, Khon Kaen, Thailand; f Department of Medicine, Nakhon Phanom Hospital, Nakhon Phanom, Thailand; g Department of Medicine, Mukdahan Hospital, Mukdahan, Thailand; h Department of Medicine, Roi Et Hospital, Roi Et, Thailand; i Department of Medicine, Surin Hospital, Surin, Thailand; j Department of Medicine, Buriram Hospital, Buriram, Thailand; k Department of Medicine, Sisaket Hospital, Sisaket, Thailand; l Department of Medicine, Srinagarind Hospital, Khon Kaen University, Khon Kaen, Thailand; m Center of Tropical Medicine and Global Health, University of Oxford, Oxford, UK; n Department of Medical Technology, Faculty of Associated Medical Science, Chiang Mai University, Chiang Mai, Thailand; o The Centre for Research and Development of Medical Diagnostic Laboratories, Khon Kaen University, Khon Kaen, Thailand; p Department of Global Health, University of Washington, Seattle, WA, USA

**Keywords:** Melioidosis, Epidemiology, Emerging infectious diseases, Neglected tropical diseases, Thailand

## Abstract

**Background:**

Melioidosis is a neglected tropical infection caused by the environmental saprophyte *Burkholderia pseudomallei.*

**Methods:**

We conducted a prospective, observational study at nine hospitals in northeastern Thailand, a hyperendemic melioidosis zone, to define current characteristics of melioidosis patients and quantify outcomes over one year.

**Findings:**

2574 individuals hospitalised with culture-confirmed melioidosis were screened and 1352 patients were analysed. The median age was 55 years, 975 (72%) were male, and 951 (70%) had diabetes. 565 (42%) patients presented with lung infection, 1042 (77%) were bacteremic, 442 (33%) received vasopressors/inotropes and 547 (40%) received mechanical ventilation. 1307 (97%) received an intravenous antibiotic against *B. pseudomallei.* 335/1345 (25%) patients died within one month and 448/1322 (34%) of patients died within one year. Most patients had risk factors for melioidosis, but patients without identified risk factors did not have a reduced risk of death. Of patients discharged alive, most received oral trimethoprim-sulfamethoxazole, which was associated with decreased risk of post-discharge death; 235/970 (24%) were readmitted, and 874/1015 (86%) survived to one year. Recurrent infection was detected in 17/994 patients (2%). Patients with risk factors other than diabetes had increased risk of death and increased risk of hospital readmission.

**Interpretation:**

In northeastern Thailand patients with melioidosis experience high rates of bacteremia, organ failure and death. Most patients discharged alive survive one year although all-cause readmission is common. Recurrent disease is rare. Strategies that emphasize prevention, rapid diagnosis and intensification of early clinical management are likely to have greatest impact in this and other resource-restricted regions.

**Funding:**

USNIH/10.13039/100000060NIAIDU01AI115520.

**Research in context**

**Evidence before this study**

We searched PubMed with the terms (“melioidosis” or “*Burkholderia pseudomallei*”) and “epidemiology” from the year 2000 to July 13, 2022. This search retrieved 712 results. These studies, almost all in English, indicated the incontrovertible presence of non-travel-associated melioidosis in south, east, and southeast Asia, Africa, the Caribbean, and central and south America. The largest studies were from Thailand and Australia and several randomised controlled trials (RCTs) indicated decreasing rates of recurrent disease; however, no large prospective multicenter studies evaluated other long-term sequelae after melioidosis. Multiple studies identified diabetes as a risk factor for melioidosis, but there were conflicting data about the association of diabetes or sulfonylurea medications with outcome.

**Added value of this study**

This prospective, multicenter, observational study provides some of the most comprehensive and novel data to date about patient characteristics and the persistently high case fatality rate of melioidosis in northeastern Thailand, where the prevalence of diabetes is increasing. The study shows that, in contrast to Australia, severity of illness and deaths from melioidosis remain unacceptably high. Most deaths occur early; patients with respiratory failure are at particular risk of death. The large majority of patients who are discharged alive following their hospitalisation survive one year although one in four will be readmitted to hospital. Recurrent infection, previously a significant concern, is now rare. While diabetes is a major risk factor for melioidosis and increasing in prevalence in Thailand, this study also demonstrates that patients with risk factors other than diabetes experience high rates of death and, in survivors, hospital readmission following a diagnosis of melioidosis.

**Implications of all the available evidence**

Melioidosis is a frequently overlooked tropical disease yet new evidence points to broader endemicity of the infection than previously recognised. In the setting of a progressive increase in diabetes in global tropical populations, melioidosis is likely to become more common. Our study indicates that outside of high-resource regions, the infection remains acutely lethal even with provision of antibiotics, source control, and organ support measures at referral hospitals. Therefore, a heightened focus on prevention, rapid diagnosis and early treatment of melioidosis is of paramount importance.

## Introduction

Melioidosis, infection caused by *Burkholderia pseudomallei*, is a public health concern throughout the tropics, especially in Southeast Asia. *B. pseudomallei* is found in soil and water, and causes infection after transcutaneous inoculation, inhalation, or ingestion.^1^ Northeastern Thailand is a hyperendemic zone of melioidosis^2^ where the burden is both substantial and expanding: the incidence has increased between 1997 and 2006 to 21 cases per 100,000 people per year^3^ and *B. pseudomallei* is the second most common cause of bacteraemia.^4^ Diabetes is the major risk factor for melioidosis and is also increasing in Thailand.^5^ A recent retrospective study in Thailand from 2012 to 2015 reported a 30 day-case fatality rate of 39%.^6^ In contrast, an Australian study reported a recent case fatality rate of 6% in the setting of prolonged intravenous treatment.^7^^,^^8^ It is likely that most cases of melioidosis worldwide occur in less well-resourced settings such as northeastern Thailand.^9^

For those who survive the initial illness, recurrent infection, either due to relapse or reinfection, is well documented. However, recurrent infection appears to be decreasing over time, perhaps related to antibiotic choice, duration, and adherence or other clinical practice changes. Recommended antibiotic regimens for melioidosis advise intravenous intensive therapy with ceftazidime or a carbapenem for at least 10–14 days (and longer depending on disease presentation) followed by at least three months of oral eradication therapy with trimethoprim-sulfamethoxazole (TMP-SMX).^1^^,^^8^ Several clinical trials in northeastern Thailand have reported one year recurrence rates of 6% in 2004, 3% in 2014, and 2% in 2018.^10^, ^11^, ^12^ While encouraging, these observations were made in the context of rigorous treatment trials that may not reflect usual practice. Additionally, little is known about other sequelae that may occur in survivors of melioidosis. Complications following hospitalisation for sepsis are common but have not been extensively studied in melioidosis.^13^

Therefore, we conducted a prospective, multi-center study with one year of follow up to define the current characteristics of melioidosis patients and prevalence of short- and long-term outcomes in northeastern Thailand.

## Methods

### Study design, participants, and outcomes

Enrollment into the study was conducted at nine hospitals in northeastern Thailand between 22 July 2015 and 31 December 2018 (Appendix, Page 3). Hospitalised patients at least 15 years of age with microbiologically-confirmed melioidosis were prospectively enrolled and followed throughout their hospital stay. Survivors were contacted at one, two, four, six, eight, ten, and twelve months after enrollment (until 31 December 2019). Clinical data were abstracted from the medical record and from the patient or surrogate decision-maker interview into a standardised case report form. Follow up interviews and systematic surveys were administered to surviving patients (Appendix, Page 3). The primary outcome measures were death at one month (defined as 28 days) and death at one year following enrollment. Secondary outcome measures were post-discharge death, readmissions to hospital, self-reported condition, and recurrent infection during the year of follow up.

### Definitions

Clinical definitions are provided in the Appendix, Page 3. Recurrent infection was defined as the second episode of a clinical sample culture growing *B. pseudomallei* after the patient received complete treatment for the first episode. If paired bacterial isolates were available, pulse-field gel electrophoresis using *SpeI* as a restriction enzyme^14^ was performed to determine whether the recurrent isolate was the same pattern as the initial isolate. If so, the recurrence was defined as relapse; otherwise, the recurrence was defined as reinfection.

### Statistical analyses

Normally distributed data are reported using mean and standard deviations. Non-normally distributed data are reported using median and interquartile range. Analyses of categorical data were performed using the Chi square or Fisher's exact tests. Analyses of continuous data were performed using the t test or rank sum tests. Information about missing data is provided in the Appendix, Page 4. Relative risks of outcomes were estimated using a modified Poisson model with robust standard errors to reduce overestimation of the error.^15^ Competing risk regression to estimate the subhazard ratio and cumulative incidence of readmission post-discharge was performed considering death as a competing risk, assuming robust standard errors. Survival curves for overall survival and post-discharge survival were compared using the log rank test. P values less than 0.05 were considered significant. Analyses were performed using Stata SE 17.0 (StataCorp, College Station, Texas, USA).

### Ethics

Informed consent was obtained from participants or their surrogate decision makers. The ethics committees of each of the nine study hospitals and the Mahidol University Faculty of Tropical Medicine approved the study (approval number MUTM 2015-002-01). The University of Washington Human Subjects Division issued a statement of non-engagement in human subjects research.

### Role of the funding source

The funding source had no role in study design, collection, analysis, or interpretation of data, or reporting of results. The corresponding authors had full access to all the data and take responsibility for the accuracy of the study.

## Results

**Fig. 1:**
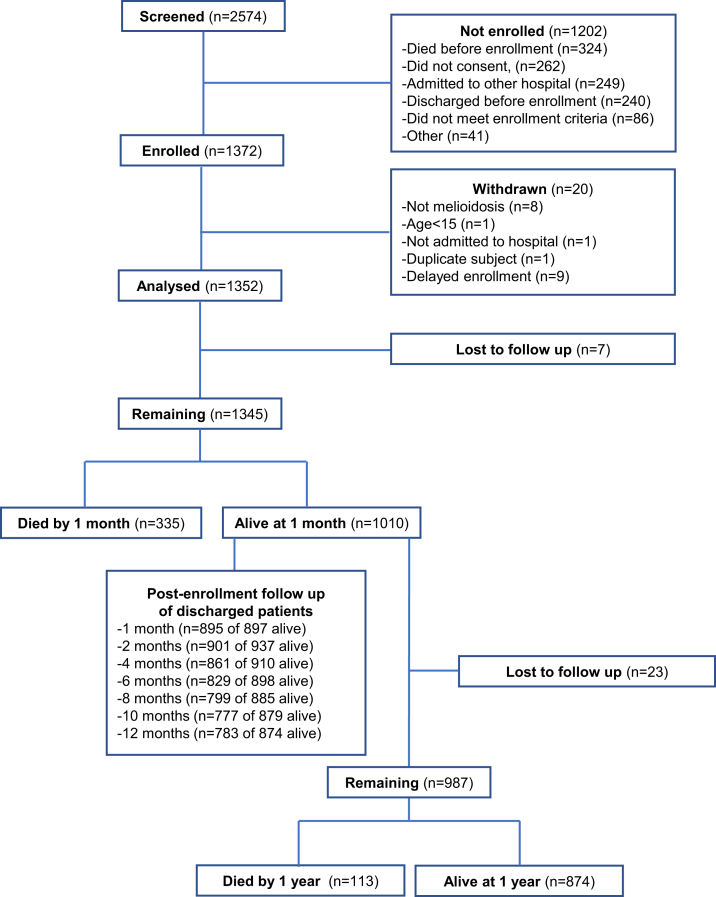
**Studyflow chart.**

2574 individuals were screened and 1372 were enrolled (Fig. 1). Twenty patients were found not to meet enrollment criteria or had delayed enrollment so were withdrawn. Therefore, 1352 patients were analysed. Median time to enrollment after admission was three days (IQR 2–4). The baseline clinical characteristics of enrolled patients are shown in Table 1. The median age was 55 years (IQR 46–64) and 975/1352 (72.1%) of patients were male. The most common pre-existing co-morbidity was diabetes, present in 951 (70.3%) of patients. While most patients had an identifiable underlying disease risk factor for acquisition of melioidosis, 177 individuals (13.1%) did not (Appendix, Page 5).^16^ Sixty four patients (4.7%) had a prior history of melioidosis (Appendix, Page 6). The most common occupation (837/1352, 61.9%) was farmer and most patients reported environmental soil (873/1352, 64.6%) or water contact (718/1352, 53.1%) most days per week. The median duration of symptoms prior to hospitalisation was 5 days (IQR 2–10).

**Table 1:** Baseline clinical characteristics of patients with melioidosis.

Patient characteristics	All (n = 1352)
Age in years – median (IQR)	55 (46–64)
Age in years, grouped – n (%)	
15-30	50 (3.7%)
31-50	436 (32.3%)
51-70	724 (53.6%)
>70	142 (10.5%)
Male – n (%)	975 (72.1%)
BMI – median (IQR)^a^	21.8 (19.5–24.1)
BMI, grouped – n (%)	
<18.5	237 (17.5%)
18.5–24.9	797 (59.0%)
25.0–29.9	193 (14.3%)
≥30.0	45 (3.3%)
Missing	80 (5.9%)
**Underlying conditions**	
Diabetes – n (%)	951 (70.3%)
Hypertension – n (%)	429 (31.7%)
Chronic kidney disease and renal calculi – n (%)	229 (16.9%)
Chronic lung disease – n (%)	164 (12.1%)
Dyslipidemia – n (%)	100 (7.4%)
Alcohol use disorder – n (%)	67 (5.0%)
Heart disease – n (%)	62 (4.6%)
Gout – n (%)	58 (4.3%)
Liver disease – n (%)	48 (3.6%)
Stroke or vascular disease – n (%)	40 (3.0%)
Cancer – n (%)	49 (3.6%)
Thalassaemia – n (%)	36 (2.7%)
Autoimmune disease – n (%)	26 (1.9%)
HIV – n (%)	24 (1.8%)
Anaemia – n (%)	18 (1.3%)
Chronic corticosteroid therapy – n (%)	51 (3.8%)
Previous melioidosis – n (%)	64 (4.7%)
No melioidosis disease risk factors – n (%)^b^	176 (13.0%)

BMI, body mass index. The number (%) of patients enrolled at each of nine sites was: 227 (16.8%), 221 (16.4%), 197 (14.6%), 178 (13.2%), 161 (11.9%), 125 (9.3%), 108 (8.0%), 105 (7.8%), and 30 (2.2%).

a BMI data are missing for 80 individuals.

b Risk factors for acquisition of melioidosis considered were diabetes, alcohol use disorder, chronic lung disease, chronic kidney disease, renal calculi, heart disease, thalassaemia, cancer, and chronic corticosteroid therapy.

841 (62.2%) patients were referred from other hospitals. Major clinical presentations are shown in Table 2 and are characterised further in the Appendix, Pages 7–9. Lung infection was the most common presentation (565, 41.8%) and the most lethal, accounting for 59.7% (200/335) of all deaths at one month. Skin and soft tissue infections were less common (307/1352, 22.7%) and were associated with lower one month mortality (42/307, 13.8%). *B. pseudomallei* bacteraemia was identified in 1042 (77.1%) patients.

**Table 2:** Major presentations of patients with melioidosis.

**Presentation**	All (n = 1352)	Bacteraemia	Septic shock	Died at one month	Died at one year
Lung infection	565 (41.8%)	430 (76.1%)	268 (47.4%)	200 (35.7%)	253 (45.8%)
Skin/soft tissue infection	307 (22.7%)	165 (53.8%)	61 (19.9%)	42 (13.8%)	57 (19.1%)
Bacteremia without focus	275 (20.3%)	275 (100.0%)	85 (30.9%)	77 (28.2%)	100 (37.3%)
Intra-abdominal infection	215 (15.9%)	184 (85.6%)	51 (23.7%)	34 (16.0%)	47 (22.5%)
Genitourinary tract infection	173 (12.8%)	153 (88.4%)	67 (38.7%)	49 (28.3%)	72 (42.4%)
Septic arthritis	108 (8.0%)	87 (80.6%)	30 (27.8%)	11 (10.3%)	23 (21.9%)
Osteomyelitis	11 (0.8%)	6 (54.6%)	2 (18.2%)	0 (0.0%)	2 (18.2%)
Neurological infection	11 (0.8%)	7 (63.6%)	3 (27.3%)	1 (9.1%)	3 (27.3%)

Sum of the first column exceeds total number of patients as some patients had more than one presentation.

Septic shock is defined as a requirement of inotropic or vasopressor agents during the hospitalization.

Vital status was missing for seven patients at one month and 30 patients at one year.

Of patients who had presented first to other hospitals, 345/841 (41.0%) received a recommended intravenous antibiotic active against *B. pseudomallei* (ceftazidime, meropenem or imipenem) prior to referral. 1307/1352 (96.7%) received an intravenous antibiotic active against *B. pseudomallei* at the study hospitals. The majority (1221; 90.3%) received ceftazidime. The median time to start these antibiotics after presentation to the study hospital was 0 days (IQR 0–2). 579 (42.8%) received TMP-SMX during their admission. 442 (32.7%) received vasopressor/intropic support and 547 (40.5%) received mechanical ventilation. 379 (28.0%) patients were cared for in an intensive care unit (ICU). 478 (35.4%) patients underwent a diagnostic, drainage or source control procedure (Appendix, Page 21). The median length of stay at the study hospital was 13 days (IQR 8–19) and was longer for survivors to hospital discharge than for those who died (14 days, IQR 9–19 vs 9 days, IQR 5–15).

**Fig. 2:**
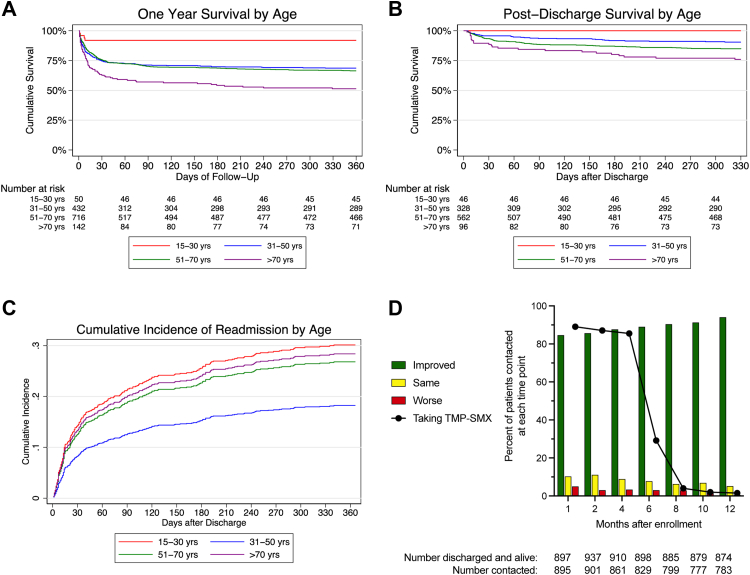
**Survival, readmission, and patient-reported clinical trajectory following melioidosis.** A: Survival of enrolled patients stratified by age. P < 0.0001 by the logrank test. B: Survival of patients discharged from hospital alive stratified by age. P < 0.0001 by the logrank test. C: Cumulative incidence of hospital readmission in patients discharged from hospital alive stratified by age. D: Overall condition since discharge and use of TMP-SMX reported by patients alive and contacted at each follow up time point. For A–C: Red line, 15–30 years; blue line, 31–50 years; green line, 51–70 years; purple line, >70 years.

Death within one month and death within one year occurred in 335 of 1345 patients (24.9%) and 448/1322 patients (33.9%) for whom vital status was known, respectively (Fig. 1). Survival curves differed significantly by age (Fig. 2A). 307/1352 (22.7%) patients died in the study hospital and 388/1019 (38.1%) surviving patients for whom disposition was known were transferred to another hospital. Of the 1010 patients alive one month after enrollment, 77 remained hospitalised at the study hospitals. 113/987 (11.5%) of patients alive one month after enrollment for whom one year vital status was known died over the subsequent 11 months; of these, 100/113 (88.5%) had been discharged from the study hospitals at the one month follow up point. Following discharge from the study hospitals, 141/1015 (13.9%) patients died during the follow up period (Fig. 2B). All-cause readmission within one year from enrollment occurred in 235 of 970 (24.2%) patients discharged alive providing data (Fig. 2C). 175/235 (74.5%) had one repeat hospitalisation; the remainder had 2-6 repeat hospitalisations. The most common reason was for treatment of infection; 43 readmissions were reported by patients as specifically relating to melioidosis (Appendix, Page 10). The median length of time to the first readmission was 34 (IQR 12–109) days (Appendix, Page 22). However, culture-proven recurrent infection was detected in only 17 of 994 (1.7%) patients.

Over 85% of discharged patients contacted at one, two, and four months post-enrollment were taking TMP-SMX (Fig. 2D). Confirmed drug reactions were rare but two to three percent reported a rash (Appendix, Page 11). Over time, the number of surviving patients who reported clinical improvement increased (Fig. 2D). At one month, 542/895 (60.6%) percent reported symptoms, most commonly fatigue, and there was a downward trajectory in symptoms reported over subsequent months (Appendix, Pages 11, 23).

Median (IQR) time to recurrence was 258 days (191–284) following enrollment. All 17 patients with culture-proven recurrent infection had received intravenous antibiotics effective against *B. pseudomallei* during their original illness. Twelve patients (70.6%) took TMP/SMX through four months of follow-up, a proportion that was statistically no different than for patients who did not experience recurrence. Of the 17 patients with recurrent infection, the recurrent bacterial isolate was obtained for 11 individuals. Ten of these patients (10/11, 90.9%) had relapsed infection; the remaining individual had reinfection with a distinct isolate. Five of the 17 patients (29.4%) with recurrent infection died within 28 days of the recurrence.

Risk factors for death within one month are shown in Table 3. In adjusted analyses, the only co-morbidities with increased risk of death were gout and liver disease. The adjusted relative risk (aRR) of death among patients presenting with lung infection was 1.80 (95% confidence interval (CI): 1.48–2.18). Patients who required vasopressor/inotropic support or mechanical ventilation had very high aRRs of death (5.84, 95% CI: 4.67–7.29, and 6.72, 95% CI: 5.10–8.85, respectively). Diabetes was associated with a decreased risk of death (aRR 0.61, 95% CI: 0.50–0.75). The absence of any identified clinical risk factor for melioidosis did not decrease the risk of death. Risk factors for death within one year were broadly similar to risk factors for death within one month although additional underlying comorbidities increased risk of mortality (Appendix, Page 12).

**Table 3:** Risk factors for death within one month after enrollment.

Patient characteristics	Survived	Died	Unadjusted RR (95% CI) P value	Adjusted RR^d^ (95% CI) P value
Age in years – n (%)^a^				
15-30	46 (92.0%)	4 (8.0%)	0.33 (0.13–0.84)	0.35 (0.14–0.88)
N = 50			P = 0.02	P = 0.03
31-50	328 (75.4%)	107 (24.6%)	Ref	Ref
N = 435			–	–
51-70	546 (76.0%)	172 (24.0%)	0.97 (0.79–1.20)	0.99 (0.80–1.22)
N = 718			P = 0.81	P = 0.93
>70	90 (63.4%)	52 (36.6%)	1.49 (1.13–1.95)	1.41 (1.08–1.85)
N = 142			P = 0.004	P = 0.01
Male – n (%)	718 (73.9%)	253 (26.1%)	1.19 (0.95–1.48)	1.18 (0.95–1.46)
N = 971			P = 0.12	P = 0.14
BMI – n (%)^b^				
<18.5	170 (71.7%)	67 (28.3%)	1.18 (0.93–1.50)	1.12 (0.89–1.41)
N = 237			P = 0.16	P = 0.33
18.5–24.9	603 (76.1%)	189 (23.9%)	Ref	Ref
N = 792			–	–
25.0–29.9	153 (79.7%)	39 (20.3%)	0.85 (0.63–1.16)	0.89 (0.65–1.20)
N = 192			P = 0.30	P = 0.43
≥30.0	34 (75.6%)	11 (24.4%)	1.02 (0.60–1.74)	1.06 (0.65–1.72)
N = 45			P = 0.93	P = 0.82
Referred from other facility – n (%)	595 (71.1%)	242 (28.9%)	1.58 (1.28–1.95)	1.46 (1.18–1.81)
N = 837			P < 0.001	P = 0.001
**Underlying conditions**				
Diabetes – n (%)	724 (76.7%)	220 (23.3%)	0.81 (0.67–0.99)	0.61 (0.50–0.75)
N = 944			P = 0.04	P < 0.001
Hypertension – n (%)	308 (72.0%)	120 (28.0%)	1.20 (0.99–1.45)	1.04 (0.85–1.27)
N = 428			P = 0.07	P = 0.69
Chronic kidney disease and renal calculi – n (%)	156 (68.4%)	72 (31.6%)	1.34 (1.08–1.67)	1.09 (0.85–1.39)
N = 228			P = 0.008	P = 0.52
Dyslipidemia – n (%)	71 (71.0%)	29 (29.0%)	1.18 (0.86–1.63)	1.24 (0.92–1.68)
N = 100			P = 0.31	P = 0.16
Chronic lung disease – n (%)	106 (64.6%)	58 (35.4%)	1.51 (1.20–1.90)	1.12 (0.87–1.44)
N = 164			P < 0.001	P = 0.38
Gout – n (%)	30 (51.7)	28 (48.3%)	2.02 (1.52–2.69)	1.73 (1.30–2.30)
N = 58			P < 0.001	P < 0.001
Liver disease – n (%)	25 (52.1%)	23 (47.9%)	1.99 (1.46–2.71)	1.54 (1.09–2.18)
N = 48			P < 0.001	P = 0.02
Heart disease – n (%)	39 (62.9%)	23 (37.1%)	1.53 (1.09–2.14)	1.05 (0.73–1.51)
N = 62			P = 0.01	P = 0.80
Stroke or vascular disease – n (%)	22 (55.0%)	18 (45.0%)	1.85 (1.30–2.64)	1.29 (0.89–1.85)
N = 40			P = 0.001	P = 0.17
Cancer – n (%)	33 (67.4%)	16 (32.7%)	1.32 (0.88–2.01)	1.30 (0.85–1.97)
N = 49			P = 0.18	P = 0.22
Alcohol use disorder – n (%)	41 (61.2%)	26 (38.8%)	1.60 (1.17–2.20)	1.23 (0.88–1.73)
N = 67			P = 0.003	P = 0.23
Thalassemia – n (%)	31 (86.1%)	5 (13.9%)	0.55 (0.24–1.25)	0.72 (0.31–1.66)
N = 36			P = 0.15	P = 0.44
Autoimmune disease – n (%)	22 (84.6%)	4 (15.4%)	0.61 (0.25–1.52)	0.72 (0.28–1.84)
N = 26			P = 0.29	P = 0.49
HIV – n (%)	15 (62.5%)	9 (37.5%)	1.52 (0.90–2.57)	1.54 (0.91–2.60)
N = 24			P = 0.12	P = 0.11
Anaemia – n (%)	14 (77.8%)	4 (22.2%)	0.89 (0.37–2.13)	0.95 (0.39–2.30)
N = 18			P = 0.80	P = 0.91
Chronic corticosteroid therapy – n (%)	35 (68.6%)	16 (31.4%)	1.27 (0.84–1.93)	1.37 (0.92–2.03)
N = 51			P = 0.26	P = 0.12
Previous melioidosis – n (%)	43 (67.2%)	21 (32.8%)	1.34 (0.93–1.93)	1.37 (0.98–1.92)
N = 64			P = 0.12	P = 0.07
No melioidosis risk factors – n (%)^c^	138 (78.4%)	38 (21.6%)	0.85 (0.63–1.15)	0.91 (0.67–1.23)
N = 176			P = 0.29	P = 0.54
**Clinical Presentations**				
Lung infection – n (%)	361 (64.4%)	200 (35.7%)	2.07 (1.71–2.50)	1.80 (1.48–2.18)
N = 561			P < 0.001	P < 0.001
Skin/soft tissue infection – n (%)	263 (86.2%)	42 (13.8%)	0.49 (0.36–0.66)	0.57 (0.42–0.77)
N = 305			P < 0.001	P < 0.001
Bacteremia without focus – n (%)	196 (71.8%)	77 (28.2%)	1.17 (0.94–1.46)	1.16 (0.94–1.45)
N = 273			P = 0.15	P = 0.17
Intra-abdominal infection – n (%)	179 (84.0%)	34 (16.0%)	0.60 (0.43–0.83)	0.65 (0.47–0.89)
N = 213			P = 0.002	P = 0.008
Genitourinary tract infection – n (%)	124 (71.7%)	49 (28.3%)	1.16 (0.90–1.50)	1.18 (0.91–1.53)
N = 173			P = 0.26	P = 0.21
Septic arthritis – n (%)	96 (89.7%)	11 (10.3%)	0.39 (0.22–0.69)	0.40 (0.22–0.71)
N = 107			P = 0.001	P = 0.002
Osteomyelitis – n (%)	11 (100.0%)	0 (0.0%)	–	–
N = 11				
Neurological infection – n (%)	10 (90.9%)	1 (9.1%)	0.36 (0.06–2.36)	0.46 (0.07–3.19)
N = 11			P = 0.29	P = 0.43
**Positive culture for *B. pseudomallei***				
Blood – n (%)	740 (71.4%)	296 (28.6%)	2.26 (1.66–3.08)	2.05 (1.51–2.79)
N = 1036			P < 0.001	P < 0.001
Respiratory tract sample – n (%)	160 (63.0%)	94 (37.1%)	1.68 (1.38–2.04)	1.47 (1.21–1.80)
N = 254			P < 0.001	P < 0.001
Urine – n (%)	41 (61.2%)	26 (38.8%)	1.60 (1.17–2.20)	1.39 (0.99–1.96)
N = 67			P = 0.003	P = 0.06
**Clinical Management**				
Received IV antibiotic active against *B. pseudomallei* – n (%)	978 (75.2%)	322 (24.8%)	0.86 (0.54–1.37)	0.92 (0.57–1.48)
N = 1300			P = 0.52	P = 0.73
Days to receipt of IV antibiotic active against *B. pseudomallei* – median (IQR)	0 (0–2)	0 (0–2)	0.97 (0.93–1.02)	0.98 (0.94–1.02)
			P = 0.22	P = 0.42
Received vasopressor/inotrope	191 (43.3%)	250 (56.7%)	6.03 (4.85–7.50)	5.84 (4.67–7.29)
N = 441			P < 0.001	P < 0.001
Received mechanical ventilation	267 (49.1%)	277 (50.9%)	7.03 (5.41–9.13)	6.72 (5.10–8.85)
N = 544			P < 0.001	P < 0.001
ICU admission – n (%)	196 (51.9%)	182 (48.2%)	3.04 (2.54–3.64)	3.00 (2.49–3.62)
N = 378			P < 0.001	P < 0.001

RR, relative risk; IQR, interquartile range; BMI, body mass index; IV, intravenous; ICU, Intensive Care Unit. Vital status at one month was not known for 7 of 1352 patients, BMI data is missing for 79 individuals, and 1300 patients received an IV antibiotic active against *B. pseudomallei* so total n = 1345 for all exposures except BMI (n = 1266) and days to receipt of IV antibiotic active against *B. pseudomallei* (n = 1300). In total, 1010 (75.1%) patients survived and 335 (24.9%) patients died at one month.

a 31–50 years is the reference group.

b 18.5–24.9 is the reference group.

c Risk factors for acquisition of melioidosis considered were diabetes, alcohol use disorder, chronic lung disease, chronic kidney disease, renal calculi, heart disease, thalassaemia, cancer, and chronic corticosteroid therapy.

d Adjusted models include age, sex, referral from other facility, time to enrollment, combined comorbidity index (except for no melioidosis risk factors model), and site as covariates.

A reduced risk of death in diabetic patients with melioidosis has been attributed to glyburide (glibencamide), a sulfonylurea medication with broad anti-inflammatory effects.^17^ In our cohort, glyburide use was rare; however, use of glyburide, glipizide, any sulfonylurea medication, or metformin was not associated with reduced risk of death among diabetic patients at one month or at one year (Appendix, Page 15). When considering specific combinations of medications (ranging from no drugs to oral agents to insulin) as proxy indicators of severity of diabetes, diabetes was associated with a reduced risk of death compared to non-diabetics across all strata of diabetes severity (Appendix, Page 15).

Patients without diabetes differed from diabetic patients by age, body mass index, and higher proportion of serious comorbidities such as chronic lung disease and cancer, suggesting that the association of diabetes with improved outcome observed may reflect more severe comorbidities predisposing to poor outcomes in non-diabetic patients (Appendix, Page 16). The risk of death at one month was the same in patients with diabetes compared to patients without any melioidosis risk factors (aRR 1.00, 95% CI: 0.73–1.36), whereas the risk of death was higher among patients with risk factors other than diabetes (aRR 1.47, 95% CI: 1.06–2.06; Table 4). A similar association was noted for one year mortality (Appendix, Pages 17, 24).

**Table 4:** Relative risk of death within one month after enrollment among patients with diabetes or other melioidosis risk factors.

Patient characteristics	Survived	Died	Unadjusted RR (95% CI) P value	Adjusted RR^a^ (95% CI) P value
No risk factors	138 (78.4%)	38 (21.6%)	Ref	Ref
N = 176			–	–
Risk factors other than diabetes	148 (65.8%)	77 (34.2%)	1.59 (1.13–2.22)	1.47 (1.06–2.06)
N = 225			P = 0.007	P = 0.02
Diabetes	724 (76.7%)	220 (23.3%)	1.08 (0.80–1.46)	1.00 (0.73–1.36)
N = 944			P = 0.62	P = 0.99

Non-diabetes risk factors for acquisition of melioidosis are alcohol use disorder, chronic lung disease, chronic kidney disease, renal calculi, heart disease, thalassaemia, cancer, and chronic corticosteroid therapy. Of 1345 patients, 1010 (75.1%) survived and 335 (24.9%) died at one month.

a Adjusted model includes age, sex, referral from other facility, time to enrollment, and site as covariates.

We assessed whether survivors of their hospitalisation had post-discharge outcomes that were associated with their risk factors, clinical presentation or with eradication therapy. Patients with risk factors other than diabetes were more likely to be readmitted than diabetics or individuals without risk factors (Appendix, Page 25). Clinical presentation was not associated with readmission. Skin/soft tissue infection was associated with reduced risk of death in crude competing risk analyses although this effect was moderated after adjustment for covariates (Appendix, Page 18). Taking TMP-SMX at one month and four months after enrollment was associated with significantly decreased risk of death at one year (aRR 0.32, 95% CI: 0.21–0.51 and aRR 0.38, 95% CI: 0.18–0.80; Appendix, Page 19).

## Discussion

The main finding of this prospective, multi-center cohort study is that melioidosis remains a highly impactful disease in northeastern Thailand. One in four enrolled patients did not survive to one month and one in three did not survive to one year. During hospitalisation, patients frequently underwent diagnostic, drainage, or source control procedures, required organ support for septic shock and respiratory failure, and were managed in intensive care units. Most deaths occurred early. Among survivors to discharge, one in four patients experienced all-cause readmissions to hospital. These results underscore the considerable burden associated with melioidosis in low resource settings and highlight stark differences in outcome compared to highly resourced hospitals.^7^ The findings suggest that strategies targeting early disease identification and management are likely to have the highest impact in mitigating this burden.

The global distribution of *B. pseudomallei* is predicted to be broader than previously recognised, yet northeastern Thailand is considered hyperendemic for melioidosis.^9^ *B. pseudomallei* is readily detectable in the soil and water^18^^,^^19^ and a recent retrospective study determined the number of melioidosis cases in the region to be at least 1300 per year.^6^ Clinicians at referral hospitals are familiar with the infection as reflected by the finding that the vast majority of patients in our study received intravenous antibiotics active against *B. pseudomallei*. There is a well-established referral system in the region for ill patients to allow them to access higher levels of care. Despite this, the case fatality rate in melioidosis remains unacceptably high. Notably, 324 patients with culture-proven melioidosis who were screened for this study died prior to enrollment. Had these patients been enrolled in our cohort, the overall one month case fatality rate would have approached 40%. Strikingly, this rate has not changed appreciably in northeastern Thailand since the introduction of ceftazidime to treat melioidosis in 1988.^20^

It is instructive to compare the results of our study with those from a recently reported single center melioidosis patient cohort from Darwin, Australia.^7^ Over the last five years, mortality from melioidosis in Darwin has decreased to 6%, over four times lower than our observed one month mortality rate of 25%. Patients in our study were more likely to be over 50 years of age, to be male, and to have diabetes. The absence of identified melioidosis disease risk factors in a minority of patients was comparable in both cohorts although the lack of identified risk factors did not reduce the risk of death in our cohort. In contrast, in the Darwin cohort, the relative risk of death among patients with no identified risk factors was 0.12 (95% CI: 0.04–0.37).^7^ Clinical presentations were largely similar and dominated by lung infection in both studies, although our cohort had a greater proportion of patients with intra-abdominal infection, most commonly liver and spleen abscesses. Across almost all disease presentations, bacteremia and septic shock were more common in our cohort.

About 40% of patients in our study had respiratory failure requiring mechanical ventilation and both septic shock and respiratory failure were very highly predictive of death. Additionally, 324 patients with melioidosis screened for the study died prior to enrollment. These data suggest that in northeastern Thailand melioidosis patients may present to referral hospitals with severe or advanced disease. Efforts focused on early identification and management of infection are therefore particularly important. Over 60% of patients were referred from other hospitals, where only a minority of patients received an intravenous antibiotic active against *B. pseudomallei*. Although we did not identify this as a risk factor for death in referred patients, maintaining high clinical suspicion for melioidosis in at risk patients (which necessitates awareness of melioidosis among front line clinicians), making a rapid diagnosis, and ensuring availability and administration of appropriate antimicrobial agents are essential tenets to improve outcomes. In addition, over one third of patients in our cohort required a surgical diagnostic or drainage procedure, capabilities that likely were less available at referring hospitals. Such procedural capacity is often an under-recognised necessity to obtain adequate source control and may be an actionable intervention to reduce mortality in severe infection and sepsis. Indeed, given the global burden of sepsis, these findings in our melioidosis cohort underscore the critical importance of continuing efforts to improve sepsis care worldwide.^21^^,^^22^

Few studies have detailed the clinical course of melioidosis patients following hospital discharge. We found that nearly one quarter of patients discharged alive required readmission to hospital over the year, and one quarter of those patients were readmitted more than once. These findings are broadly concordant with studies in highly resourced settings indicating that readmission following hospitalisation for sepsis or pneumonia is common.^23^, ^24^, ^25^ Most readmissions occurred within the early weeks after discharge indicating that this time frame may be a potential target for future interventions to reduce the need for rehospitalisation. These readmissions may not be directly related to melioidosis; they may instead reflect the frailty of individuals who acquire melioidosis, given the high frequency of comorbidities and disease risk factors in the population. Together, these observations indicate that surviving melioidosis patients are at high risk of further complications and require close follow up. Australian guidelines now suggest prolonged courses of intravenous therapy for specific disease presentations.^8^ Given that most patients in this study were likely to receive shorter courses of intravenous antibiotics, at least as inpatients at the study sites, adoption of these approaches may have benefit in reducing hospital readmissions and late complications.

The majority of patients in this cohort had diabetes, the main risk factor for melioidosis. Yet, to our knowledge the observed proportion of diabetics (70%) is one of the highest observed in any large cohort study of melioidosis to date. This is comparable to the proportion of diabetics observed in a recently reported randomised controlled clinical trial of melioidosis treatment in northeastern Thailand.^12^ In contrast two trials in the region that concluded in 2003 reported 59% of patients with diabetes.^26^ This points to an even greater role for diabetes in driving the number of melioidosis cases than previously noted. In line with this observation, the prevalence of diabetes in Thailand and throughout the world is increasing.^5^^,^^27^ This may be due at least in part to dietary changes, physical activity patterns, and urbanisation.^5^ Moreover, much diabetes in Thailand is undiagnosed and even among patients with known disease, glycemic control is worse in the melioidosis-endemic northeastern region of the country.^28^ These observations have worrisome implications for the burden of melioidosis not only in Thailand but around the world. For example, south Asia, where melioidosis is being increasingly identified, is home to nearly two billion people with a prevalence of diabetes of about eight percent.^9^^,^^29^

The association of diabetes with reduced risk of death is apparently paradoxical given that diabetes is the major risk factor for acquisition of melioidosis. However, prior studies have also reported that, among patients with melioidosis, diabetics have lower rates of death.^6^^,^^7^^,^^17^ One proposed explanation is the anti-inflammatory effect of the oral sulfonylurea glyburide (glibencamide).^17^ We did not find that glyburide alone or sulfonylurea therapy was associated with a reduced risk of death; however, we may not have had adequate power to detect an effect of glyburide because few patients were taking this medication. Another possible explanation is that patients without diabetes have risk factors that put them at increased risk of death. We determined that diabetes does not alter risk of hospital readmission or death compared to non-diabetics without other melioidosis risk factors. In contrast, non-diabetic patients with other risk factors for disease have significantly increased risks of readmission or death. This finding argues against any protective effect of diabetes and instead reflects the fact that melioidosis is an opportunistic infection that causes poorer outcomes in individuals with more severe comorbidities.^7^

Failure to achieve complete eradication of *B. pseudomallei* has been a long-standing concern in melioidosis. Following the intensive intravenous regimen, oral TMP-SMX therapy is currently recommended for 12 weeks.^12^ We found that TMP-SMX therapy following hospital discharge was associated with decreased risk of death. Past randomised controlled clinical trials have reported one year recurrence rates that have decreased from 6% in 2004 to 2% in 2020 but these observed rates in clinical trials may not reflect actual practice.^10^, ^11^, ^12^ Our large observational cohort confirmed that culture-confirmed recurrent infection, despite active surveillance, was comparably rare at 1.7%. Of the recurrent cases, as in previous studies,^30^ most were relapsed infection. We did not identify any overt failures in antibiotic selection or adherence, risk factors for relapse.^30^ Our observed recurrence rate parallels that from Australia^31^ and probably reflects increasing local knowledge of melioidosis management.

Over 60% of patients in our study were farmers and the majority of patients were exposed to soil and water on a regular basis, reflecting likely sources of infection. In the absence of a melioidosis vaccine, evidence-based guidelines such as wearing boots and drinking bottled water to reduce the risk of infection have been developed.^32^ While prevention efforts are essential to reduce the burden of disease,^33^ a multifaceted behavioural intervention in the northeastern Thailand did not demonstrate effectiveness in reducing the incidence of culture-confirmed melioidosis in diabetics.^34^

Strengths of this study are its prospective, multi-center design, size, and long duration and high rates of follow up. We estimate that we enrolled roughly one quarter of all melioidosis patients in northeastern Thailand during the time frame of our study.^6^ Limitations include the delay in enrolling patients until confirmed melioidosis, thus missing patients who died or were discharged early. We did not capture detailed management data from non-study hospitals. Our models may be impacted by unmeasured confounders, especially with regard to unappreciated risk factors for poor outcomes that may bias our results towards the null hypothesis. Our study was not designed to include a comparator cohort without melioidosis to permit the attribution of risk of melioidosis on outcomes nor do we have data about participants’ symptoms prior to infection. As our study was performed entirely in northeastern Thailand, our results may not be generalisable elsewhere.

In conclusion, in northeastern Thailand patients hospitalised with melioidosis experience high rates of bacteremia, organ failure and early death. While most patients are diabetics, those with other risk factors for infection are at highest risk of poor outcomes. Most patients discharged alive survive through one year although readmission is common. Recurrent disease is rare. In addition to prevention efforts, strategies that accelerate rapid diagnosis and intensify early clinical management of melioidosis, including prior to referral, are likely to have greatest impact in this region and in other resource-restricted settings.

## Contributors

NC and TEW obtained funding, designed the study and provided supervision. RP, AY, AD, TY, ET, SC, KT, SC, CM, NS, SC, WS, NB, PC and GL contributed to data collection. TEW, RP, LO and NC performed data analysis and interpretation. RP, NC, and TEW accessed and verified the data. TEW and NC wrote the manuscript. NC, RP, AY, AD, TY, LO, ET, SC, KT, SC, CM, NS, SC, WS, NB, PC, NPJD, GL and TEW reviewed the manuscript.

## Data sharing statement

Following publication, summary data, case report forms, and consent forms from this study are available upon request from the authors.

## Declaration of interests

The project was funded by 10.13039/100000002NIH/10.13039/100000060NIAID award U01AI115520 to Narisara Chantratita and T. Eoin West.

The authors declare no competing interests.
