# Supplementary material for: Characteristics and one year outcomes of melioidosis patients in Northeastern Thailand: a prospective, multicenter cohort study
Source: Lancet Reg Health Southeast Asia. 2022 Nov 25;9:100118. doi: 10.1016/j.lansea.2022.100118 (PMC9788505; doi:10.1016/j.lansea.2022.100118)
Supplement: Supplementary File S1 [file mmc1.pdf]

APPENDIX

Characteristics and One Year Outcomes of Melioidosis Patients in Northeastern Thailand: A Prospective, Multicenter Cohort Study

Prof Narisara Chantratita, PhD[1,2*], Rungnapa Phunpang, BNS[1,2], Atchara Yarasai, MA[1], Adul Dulsuk, BSc[1,2], Thatcha Yimthin, MSc[1], Lauren A. Onofrey, MD[3], Taylor D. Coston, MD[3], Ekkachai Thiansukhon, MD[4], Seksan Chaisuksant, MD[5], Kittisak Tanwisaid, MD[6], Somchai Chuananont, MD[6], Chumpol Morakot, MD[7], Narongchai Sangsa, MD[8], Sunee Chayangsu, MD[9], Wirayut Silakun, MD[10], Noppol Buasi, MD[11], Prof Ploenchan Chetchotisakd, MD[12], Prof Nicholas P.J. Day, MD[2,13], Ganjana Lertmemongkolchai, PhD[14,15], Prof T. Eoin West, MD[1,3,16*]

[1]Department of Microbiology and Immunology, Faculty of Tropical Medicine, Mahidol University, Bangkok, Thailand
[2]Mahidol-Oxford Tropical Medicine Research Unit, Faculty of Tropical Medicine, Mahidol University, Bangkok, Thailand
[3]Division of Pulmonary, Critical Care & Sleep Medicine, Department of Medicine, University of Washington, Seattle, Washington, USA
[4]Department of Medicine, Udon Thani Hospital, Udon Thani, Thailand
[5]Department of Medicine, Khon Kaen Regional Hospital, Khon Kaen, Thailand
[6]Department of Medicine, Nakhon Phanom Hospital, Nakhon Phanom, Thailand
[7]Department of Medicine, Mukdahan Hospital, Mukdahan, Thailand
[8]Department of Medicine, Roi Et Hospital, Roi Et, Thailand
[9]Department of Medicine, Surin Hospital, Surin, Thailand
[10]Department of Medicine, Buriram Hospital, Buriram, Thailand
[11]Department of Medicine, Sisaket Hospital, Sisaket, Thailand
[12]Department of Medicine, Srinagarind Hospital, Khon Kaen University, Khon Kaen, Thailand
[13]Center of Tropical Medicine and Global Health, University of Oxford, Oxford, UK
[14]Department of Medical Technology, Faculty of Associated Medical Science, Chiang Mai University, Chiang Mai, Thailand
[15]The Centre for Research and Development of Medical Diagnostic Laboratories, Khon Kaen University, Khon Kaen, Thailand
[16]Department of Global Health, University of Washington, Seattle, Washington, USA

Table of Contents

Supplemental Methods Page 3

Table S1. Characteristics of patients with and without established melioidosis risk factors Page 5

Table S2. Characteristics of patients with or without prior melioidosis Page 6

Table S3. Characteristics of patients with or without lung infection Page 7

Table S4. Characteristics of patients with or without skin/soft tissue infection Page 8

Table S5. Characteristics of patients with or without bacteraemia without a focus Page 9

Table S6. Reasons for readmission among patients discharged from hospital alive Page 10

Table S7. Percent of patients experiencing symptoms after hospital discharge Page 11

Table S8. Risk factors for death within one year after enrollment Page 12

Table S9. Diabetic therapies and relative risk of death at one month and at one year Page 15

Table S10. Characteristics of melioidosis patients with or without diabetes Page 16

Table S11. Relative risk of death within one year after enrollment among patients with diabetes or other melioidosis risk factors Page 17

Table S12. Relative risk of post-discharge death within one year after enrollment among patients with specific clinical presentations Page 18

Table S13. Relative risk of post-discharge death within one year after enrollment among patients taking TMP-SMX eradication therapy Page 19

Figure Legends Page 20

Figure S1. Diagnostic and therapeutic drainage procedures performed Page 21

Figure S2. Time to readmission after discharge Page 22

Figure S3. Symptoms reported following hospital discharge Page 23

Figure S4. One year survival by melioidosis risk factors Page 24

Figure S5. Cumulative incidence of hospital readmission by melioidosis risk factors Page 25

Data Sharing Page 26

Supplemental Methods

Study design

The study was conducted at the following hospitals in northeastern Thailand: Udon Thani Hospital, Khon Kaen Hospital, Srinagarind Hospital, Nakhon Phanom Hospital, Mukdahan Hospital, Roi Et Hospital, Buriram Hospital, Surin Hospital, and Sisaket Hospital. All nine study hospitals had ICU availability and surgical care, and offered the following organ support capabilities: intravenous inotropes/vasopressors, mechanical ventilation, and renal replacement therapy. A target study size of 1400 patients was initially determined based on an estimated relapsed infection rate of 4.5% among survivors to hospital discharge for investigation of biological determinants of relapse. The diagnosis of melioidosis was determined by culture of an organism that was identified as *B. pseudomallei* using standard methodologies and/or Vitek 2, a positive latex agglutination test (Duval BD et al , Am J Trop Med Hyg, 2014), or (at Udon Thani Hospital) immunofluorescence microscopy assay (Chantratita N al, Am J Trop Med Hyg, 2013; Dulsuk A et al, Trans R Soc Trop Med Hyg, 2016). Pregnancy, receipt of palliative care, or incarceration were exclusion criteria.

Patient follow up interviews

Interviews were conducted with surviving patients discharged from the study hospitals at 1, 2, 4, 6, 8, 10, and 12 months following enrollment. At each time point, systematic surveys were administered using a standardized case report form and inquired about self-reported overall condition as well as presence or absence of specific symptoms experienced since the previous encounter. Current medications, new diagnoses, any hospital readmissions, and reasons for readmission were recorded.

Definitions

All pre-existing conditions were determined based on medical diagnoses entered in the chart by treating clinicians. The definition of diabetes in Thailand is based on national Thai guidelines (www.diabassocthai.org), including hemoglobin A1c ≥6·5% or a random blood glucose ≥11·1 mmol/L (200mg/dl) in the presence of signs and symptoms. The generally accepted definition of CKD is a glomerular filtration rate less than 60 ml/min/1·73 m^2 or markers of kidney damage. Chronic lung disease included COPD, bronchiectasis, asthma, and previous tuberculosis. Autoimmune disease was defined as rheumatoid arthritis or systemic lupus erythematosus. Alcohol use disorder was based on clinicians' diagnoses or a history of routinely consuming more than two alcoholic beverages daily. Chronic corticosteroid therapy was defined as taking more than or equal to the equivalent of five milligrams of prednisone daily. A combined comorbidity index was calculated by assigning one point for a history of the following conditions: diabetes, heart disease, chronic kidney disease, chronic lung disease, cancer, liver disease, stroke or vascular disease, HIV/AIDS, autoimmune disease, or alcohol use disorder. Previous melioidosis was defined as a diagnosis of melioidosis at least four months prior to the current hospitalisation.

Presentations of disease during the index hospitalisation were defined using admission and discharge diagnoses made by treating clinicians, and using microbiological data as follows: Lung infection included diagnoses of pneumonia (non-nosocomial and non-attributable to other pathogens), lung abscess, suspected TB (on admission only), empyema, or the culture of *B. pseudomallei* from a respiratory tract sample. Skin/soft tissue infection included diagnoses of cellulitis, infected wounds or hematomas, abscesses of soft tissues, myositis, fasciitis, and gangrene. Intra-abdominal infection included peritoneal and omental infection, and liver and spleen abscesses. Genitourinary infection included urinary tract infection, cystitis, pyelonephritis, prostate, testicular or ovarian abscesses that were not attributable to other pathogens as well as *B. pseudomallei* bacteriuria complicating renal calculi. Septic arthritis and osteomyelitis categories included these specific conditions. Neurological infection included meningitis, encephalitis, brain abscess, and epidural abscess. Bacteremia without a focus included patients with bacteremia who did not have an evident site of infection elsewhere. Other than bacteremia without a focus, categories were not mutually exclusive.

Discharge was defined as discharge from one of the study sites although some patients were transferred to other hospitals. Due to the common practice of discharging terminally ill patients to die at home at their request, patients who died within three days after hospital discharge were considered to have died in hospital.

Missing data

Prior to analysis, the dataset was examined for missing data. There were no missing data for age or sex. Comorbidity and clinical management data were not available for one patient during hospitalisation; for this individual

comorbidities or clinical interventions were considered absent or not performed. Not all patients had cultures of all body fluids performed or recorded. It was therefore assumed that cultures were performed where possible and absent culture results were treated as negative. One month vital status was not available for seven patients; these patients were not included in analyses of one month mortality. One year vital status was not available for thirty patients; these patients were not included in analyses of one year mortality. Post-discharge followup analyses were performed using the number of patients contacted at each time point as the denominator.

Statistical Analysis

In all models, interactions between age and sex were investigated but not identified. Therefore, the models reported do not include age and sex interaction terms.

Table S1. Characteristics of patients with and without established melioidosis risk factors

Patient characteristics	Risk factors* (n = 1176)	No risk factors (n = 176)	P value
Age in years, grouped – n (%)			
15-30	33 (2·8%)	17 (9·7%)	<0·001
31-50	386 (32·8%)	50 (28·4%)	
51-70	635 (54·0%)	89 (50·6%)	
>70	122 (10·4%)	20 (11·4%)	
Male – n (%)	837 (71·2%)	138 (78·4%)	0·05
BMI, grouped – n (%)**			
<18·5	201 (17·1%)	36 (20·5%)	0·30
18·5-24·9	689 (58·6%)	108 (61·4%)	
25·0-29·9	176 (15·0%)	17 (9·7%)	
≥30·0	41 (3·5%)	4 (2·3%)	
**Underlying conditions**			
Hypertension – n (%)	406 (34·5%)	23 (13·1%)	<0·001
Dyslipidemia – n (%)	94 (8·0%)	6 (3·4%)	0·03
Gout – n (%)	49 (4·2%)	9 (5·1%)	0·55
Liver disease – n (%)	45 (3·8%)	3 (1·7%)	0·19
Stroke or vascular disease – n (%)	35 (3·0%)	5 (2·8%)	1·00
Autoimmune disease – n (%)	22 (1·9%)	4 (2·3%)	0·77
HIV – n (%)	15 (1·3%)	9 (5·1%)	0·002
Anemia – n (%)	15 (1·3%)	3 (1·7%)	0·72
**Clinical presentation and microbiology**			
Lung infection – n (%)	503 (42·8%)	63 (35·2%)	0·06
Skin/soft tissue infection – n (%)	252 (21·4%)	55 (31·3%)	0·004
Bacteremia without focus – n (%)	245 (20·8%)	30 (17·0%)	0·24
Intra-abdominal infection– n (%)	198 (16·8%)	17 (9·7%)	0·02
Genitourinary tract infection – n (%)	143 (12·2%)	30 (17·0%)	0·07
Septic arthritis – n (%)	96 (8·2%)	12 (6·8%)	0·54
Osteomyelitis – n (%)	10 (0·9%)	1 (0·6%)	1·00
Neurological infection – n (%)	7 (0·6%)	4 (2·3%)	0·04
Bacteremia – n (%)	926 (78·7%)	116 (65·9%)	<0·001
**Clinical outcome**			
Death at 1 month – n (%)	297 (25·4%)	38 (21·6%)	0·28
Death at 1 year – n (%)	396 (34·4%)	52 (30·4%)	0·30

* Risk factors for acquisition of melioidosis considered were diabetes, alcohol use disorder, chronic lung disease, chronic kidney disease, renal calculi, heart disease, thalassaemia, cancer, and chronic corticosteroid therapy.
** BMI data are missing for 80 patients.

Table S2. Characteristics of patients with or without prior melioidosis

Patient characteristics	No prior melioidosis (n = 1288)	Prior melioidosis (n = 64)	P value
Age in years, grouped – n (%)			
15-30	49 (3·8%)	1 (1·6%)	0·35
31-50	412 (32·0%)	24 (37·5%)	
51-70	688 (53·4%)	36 (56·3%)	
>70	139 (10·8%)	3 (4·7%)	
Male – n (%)	928 (72·1%)	47 (73·4%)	0·81
BMI, grouped – n (%)*			
<18·5	225 (17·5%)	12 (18·8%)	0·93
18·5-24·9	760 (59·0%)	37 (57·8%)	
25·0-29·9	184 (14·3%)	9 (14·1%)	
≥30·0	42 (3·3%)	3 (4·7%)	
**Underlying conditions**			
Diabetes – n (%)	897 (69·6%)	54 (84·4%)	0·01
Hypertension – n (%)	407 (31·6%)	22 (34·4%)	0·64
Chronic kidney disease and renal calculi – n (%)	220 (17·1%)	9 (14·1%)	0·61
Dyslipidemia – n (%)	95 (7·4%)	5 (7·8%)	0·81
Lung disease – n (%)	152 (11·8%)	12 (18·8%)	0·10
Gout – n (%)	56 (4·4%)	2 (3·1%)	1·00
Liver disease – n (%)	43 (3·3%)	5 (7·8%)	0·07
Heart disease – n (%)	61 (4·7%)	1 (1·6%)	0·36
Stroke or vascular disease – n (%)	38 (3·0%)	2 (3·1%)	0·71
Cancer – n (%)	47 (3·7%)	2 (3·1%)	1·00
Alcohol use disorder – n (%)	63 (4·9%)	4 (6·3%)	0·55
Thalassemia – n (%)	33 (2·6%)	3 (4·7%)	0·24
Autoimmune disease – n (%)	24 (1·9%)	2 (3·1%)	0·35
HIV – n (%)	22 (1·7%)	2 (3·1%)	0·32
Anemia – n (%)	18 (1·4%)	0 (0%)	1·00
Chronic corticosteroid therapy – n (%)	46 (3·6%)	5 (7·8%)	0·09
No melioidosis risk factors – n (%)**	175 (13·6%)	1 (1·6%)	0·002

* BMI data are missing for 80 patients.

** Risk factors for acquisition of melioidosis considered were diabetes, alcohol use disorder, chronic lung disease, chronic kidney disease, renal calculi, heart disease, thalassaemia, cancer, and chronic corticosteroid therapy.

Table S3. Characteristics of patients with or without lung infection

Patient characteristics	No lung infection (n = 787)	Lung infection (n = 565)	P value
Age in years, grouped – n (%)			
15-30	34 (4·3%)	16 (2·8%)	0·23
31-50	252 (32·0%)	184 (32·6%)	
51-70	427 (54·3%)	297 (52·6%)	
>70	74 (9·4%)	68 (12·0%)	
Male – n (%)	557 (70·8%)	418 (74·0%)	0·20
BMI, grouped – n (%)*			
<18·5	114 (15·3%)	123 (23·4%)	<0·001
18·5-24·9	467 (62·5%)	330 (62·9%)	
25·0-29·9	134 (17·9%)	59 (11·2%)	
≥30·0	32 (4·3%)	13 (2·5%)	
**Underlying conditions**			
Diabetes – n (%)	553 (70·3%)	398 (70·4%)	0·94
Hypertension – n (%)	259 (32·9%)	170 (30·1%)	0·27
Chronic kidney disease and renal calculi – n (%)	138 (17·5%)	91 (16·1%)	0·49
Dyslipidemia – n (%)	61 (7·8%)	39 (6·9%)	0·56
Chronic lung disease – n (%)	44 (5·6%)	120 (21·2%)	<0·001
Gout – n (%)	38 (4·8%)	20 (3·5%)	0·25
Liver disease – n (%)	30 (3·8%)	18 (3·2%)	0·54
Heart disease – n (%)	34 (4·3%)	28 (5·0%)	0·58
Stroke or vascular disease– n (%)	18 (2·3%)	22 (3·9%)	0·09
Cancer – n (%)	32 (4·1%)	17 (3·0%)	0·31
Alcohol use disorder – n (%)	35 (4·5%)	32 (5·7%)	0·31
Thalassaemia – n (%)	30 (3·8%)	6 (1·1%)	0·002
Autoimmune disease – n (%)	16 (2·0%)	10 (1·8%)	0·73
HIV – n (%)	10 (1·3%)	14 (2·5%)	0·10
Anaemia – n (%)	11 (1·4%)	7 (1·2%)	1·00
Chronic corticosteroid therapy – n (%)	30 (3·8%)	21 (3·7%)	0·93
Previous melioidosis – n (%)	33 (4·2%)	31 (5·5%)	0·27
No melioidosis disease risk factors – n (%)**	114 (14·5%)	62 (11·0%)	0·06

* BMI data are missing for 80 individuals.
** Risk factors for acquisition of melioidosis considered were diabetes, alcohol use disorder, chronic lung disease, chronic kidney disease, renal calculi, heart disease, thalassaemia, cancer, and chronic corticosteroid therapy.

Table S4. Characteristics of patients with or without skin/soft tissue infection

Patient characteristics	No SSTI (n = 1045)	SSTI (n = 307)	P value
Age in years, grouped – n (%)			
15-30	34 (3·3%)	16 (5·2%	0·08
31-50	337 (32·3%)	99 (32·3%)	
51-70	554 (53·0%)	170 (55·4%)	
>70	120 (11·5%)	22 (7·2%)	
Male – n (%)	767 (73·4%)	208 (67·8%)	0·05
BMI, grouped – n (%)*			
<18·5	209 (21·4%)	28 (9·6%)	<0·001
18·5-24·9	618 (63·1%)	179 (61·1%)	
25·0-29·9	128 (13·1%)	65 (22·2%)	
≥30·0	24 (2·5%)	21 (7·2%)	
**Underlying conditions**			
Diabetes – n (%)	732 (70·1%)	219 (71·3%)	0·66
Hypertension – n (%)	322 (30·8%)	107 (34·9%)	0·18
Chronic kidney disease and renal calculi – n (%)	191 (18·3%)	38 (12·4%)	0·02
Dyslipidemia – n (%)	71 (6·8%)	29 (9·5%)	0·12
Chronic lung disease – n (%)	143 (13·7%)	21 (6·8%)	0·001
Gout – n (%)	42 (4·0%)	16 (5·2%)	0·37
Liver disease – n (%)	40 (3·8%)	8 (2·6%)	0·38
Heart disease – n (%)	54 (5·2%)	8 (2·6%)	0·06
Stroke or vascular disease – n (%)	33 (3·2%)	7 (2·3%)	0·57
Cancer – n (%)	42 (4·0%)	7 (2·3%)	0·17
Alcohol use disorder – n (%)	57 (5·5%)	10 (3·3%)	0·12
Thalassaemia – n (%)	28 (2·7%)	8 (2·6%)	1·00
Autoimmune disease – n (%)	20 (1·9%)	6 (2·0%)	1·00
HIV – n (%)	21 (2·0%)	3 (1·0%)	0·33
Anaemia – n (%)	14 (1·3%)	4 (1·3%)	1·00
Chronic corticosteroid therapy – n (%)	40 (3·8%)	11 (3·6%)	0·84
Previous melioidosis – n (%)	46 (4·4%)	18 (5·9%)	0·29
No melioidosis disease risk factors – n (%)**	121 (11·6%)	55 (17·9%)	0·004

SSTI: skin/soft tissue infection.
* BMI data were missing for 80 individuals.
** Risk factors for acquisition of melioidosis considered were diabetes, alcohol use disorder, chronic lung disease, chronic kidney disease, renal calculi, heart disease, thalassaemia, cancer, and chronic corticosteroid therapy.

Table S5. Characteristics of patients with or without bacteraemia without a focus

Patient characteristics	No bacteraemia without a focus (n = 1077)	Bacteraemia without a focus (n = 275)	P value
Age in years, grouped – n (%)			
15-30	44 (4·1%)	6 (2·2%)	0·13
31-50	357 (33·2%)	79 (28·7%)	
51-70	569 (52·8%)	155 (56·4%)	
>70	107 (9·9%)	35 (12·7%)	
Male – n (%)	780 (72·4%)	195 (70·9%)	0·62
BMI, grouped – n (%)*			
<18·5	196 (19·3%)	41 (16·1%)	0·64
18·5-24·9	630 (61·9%)	167 (65·8%)	
25·0-29·9	155 (15·2%)	38 (15·0%)	
≥30·0	37 (3·6%)	8 (3·2%)	
**Underlying conditions**			
Diabetes – n (%)	764 (70·9%)	187 (68·0%)	0·34
Hypertension – n (%)	339 (31·5%)	90 (32·7%)	0·69
Chronic kidney disease and renal calculi – n (%)	172 (16·0%)	57 (20·7%)	0·06
Dyslipidemia – n (%)	76 (7·1%)	24 (8·7%)	0·35
Chronic lung disease – n (%)	142 (13·2%)	22 (8·0%)	0·02
Gout – n (%)	45 (4·2%)	13 (4·7%)	0·69
Liver disease – n (%)	32 (3·0%)	16 (5·8%)	0·02
Heart disease – n (%)	42 (3·9%)	20 (7·3%)	0·02
Stroke or vascular disease – n (%)	32 (3·0%)	8 (2·9%)	1·00
Cancer – n (%)	32 (3·0%)	17 (6·2%)	0·01
Alcohol use disorder – n (%)	47 (4·4%)	20 (7·3%)	0·05
Thalassaemia – n (%)	27 (2·5%)	9 (3·3%)	0·53
Autoimmune disease – n (%)	22 (2·0%)	4 (1·5%)	0·63
HIV – n (%)	20 (1·9%)	4 (1·5%)	0·80
Anaemia – n (%)	14 (1·3%)	4 (1·5%)	0·77
Chronic corticosteroid therapy – n (%)	41 (3·8%)	10 (3·6%)	0·90
Previous melioidosis – n (%)	57 (5·3%)	7 (2·6%)	0·06
No melioidosis disease risk factors – n (%)**	146 (13·6%)	30 (10·9%)	0·24

* BMI data were missing for 80 individuals.
** Risk factors for acquisition of melioidosis considered were diabetes, alcohol use disorder, chronic lung disease, chronic kidney disease, heart disease, thalassaemia, cancer, and chronic corticosteroid therapy.

Table S6. Reasons for readmission among patients discharged from hospital alive

Reason	n (%)
Any infection	141 (34·3%)
Melioidosis	43 (10·5%)
Gastrointestinal disorder	34 (8·3%)
Respiratory disorder	31 (7·5%)
Anemia	24 (5·8%)
Hypo- or hyperglycemia	18 (4·4%)
Fatigue	10 (2·4%)
Acute kidney injury	8 (1·9%)
Seizure	6 (1·5%)
Complication of CAPD	5 (1·2%)
Thrombocytopenia	4 (1·0%)
Stroke	2 (0·5%)
Other	85 (20·7%)

Sum of all rows (n=411) exceeds total number of readmissions (n=320) as there were more reasons than readmissions.

Table S7. Percent of patients experiencing symptoms after hospital discharge

	1 month	2 months	4 months	6 months	8 months	10 months	12 months
**Number contacted***	895	901	861	829	799	777	783
**Overall status since discharge****							
Improved	758 (84·7%)	772 (85·7%)	755 (87·7%)	738 (89·0%)	722 (90·4%)	709 (91·2%)	737 (94·1%)
Same	92 (10·3%)	101 (11·2%)	77 (8·9%)	65 (7·8%)	50 (6·3%)	54 (6·9%)	41 (5·2%)
Worse	45 (5·0%)	28 (3·1%)	29 (3·4%)	26 (3·1%)	23 (2·9%)	12 (1·5%)	5 (0·6%)
**Symptoms**							
Any symptoms**	542 (60·6%)	435 (48·3%)	346 (40·2%)	285 (34·4%)	225 (28·2%)	207 (26·6%)	181 (23·1%)
Constitutional							
Fever	74 (8·3%)	38 (4·2%)	32 (3·7%)	28 (3·4%)	20 (2·5%)	20 (2·6%)	14 (1·8%)
Fatigue	306 (34·2%)	222 (24·6%)	149 (17·3%)	119 (14·4%)	88 (11·0%)	69 (8·9%)	58 (7·4%)
Generalized weakness	36 (4·0%)	36 (4·0%)	25 (2·9%)	22 (2·7%)	16 (2·0%)	21 (2·7%)	15 (1·9%)
Weight loss	17 (1·9%)	7 (0·8%)	8 (0·9%)	3 (0·4%)	3 (0·4%)	2 (0·3%)	0 (0·0%)
Pain	139 (15·5%)	129 (14·3%)	107 (12·4%)	91 (11·0%)	68 (8·5%)	59 (7·6%)	45 (5·7%)
Musculoskeletal							
Muscle ache	11 (1·2%)	14 (1·6%)	12 (1·4%)	9 (1·1%)	8 (1·0%)	7 (0·9%)	4 (0·5%)
Joint swelling	8 (0·9%)	8 (0·9%)	2 (0·2%)	2 (0·2%)	5 (0·6%)	2 (0·3%)	1 (0·1%)
Localized swelling	25 (2·8%)	19 (2·1%)	17 (2·0%)	19 (2·3%)	11 (1·4%)	7 (0·9%)	3 (0·4%)
Neurological							
Headache	17 (1·9%)	8 (0·9%)	10 (1·2%)	6 (0·7%)	7 (0·9%)	6 (0·8%)	4 (0·5%)
Localized weakness	12 (1·3%)	13 (1·4%)	11 (1·3%)	9 (1·1%)	12 (1·5%)	12 (1·5%)	10 (1·3%)
Localized numbness	21 (2·3%)	32 (3·6%)	37 (4·3%)	30 (3·6%)	31 (3·9%)	30 (3·9%)	23 (2·9%)
Gastrointestinal							
Vomiting	34 (3·8%)	20 (2·2%)	16 (1·9%)	12 (1·4%)	4 (0·5%)	8 (1·0%)	4 (0·5%)
Diarrhea	15 (1·7%)	3 (0·3%)	1 (0·1%)	5 (0·6%)	2 (0·3%)	7 (0·9%)	1 (0·1%)
Poor appetite	56 (6·3%)	39 (4·3%)	25 (2·9%)	20 (2·4%)	14 (1·8%)	8 (1·0%)	2 (0·3%)
Jaundice	5 (0·6%)	2 (0·2%)	2 (0·2%)	2 (0·2%)	1 (0·1%)	1 (0·1%)	1 (0·1%)
Hematological							
Bleeding	5 (0·6%)	2 (0·2%)	1 (0·1%)	0 (0·0%)	2 (0·3%)	1 (0·1%)	1 (0·1%)
Respiratory							
Cough	62 (6·9%)	43 (4·8%)	37 (4·3%)	43 (5·2%)	26 (3·3%)	22 (2·8%)	18 (2·3%)
Shortness of breath	7 (0·8%)	11 (1·2%)	8 (0·9%)	12 (1·4%)	9 (1·1%)	6 (0·8%)	3 (0·4%)
Urological							
Pain on urination	4 (0·4%)	6 (0·7%)	5 (0·6%)	2 (0·2%)	2 (0·3%)	1 (0·1%)	0 (0·0%)
Dematological							
Skin rash	13 (1·5%)	25 (2·8%)	21 (2·4%)	10 (1·2%)	7 (0·9%)	8 (1·0%)	4 (0·5%)
Other	143 (16·0%)	133 (14·8%)	107 (12·4%)	92 (11·1%)	63 (7·9%)	66 (8·5%)	65 (8·3%)

* The number of patients who had left hospital and were alive at the indicated follow up times were: 1 month: 897, 2 months: 937, 4 months: 910; 6 months: 898; 8 months: 885; 10 months: 879; 12 months 874.
** All numbers are n (%) of the number of patients contacted at each follow up time.

Table S8. Risk factors for death within one year after enrollment

Patient characteristics	Survived	Died	Unadjusted RR (95% CI) P value	Adjusted RR*** (95% CI) P value
Age in years – n (%)				
15-30 N=49	45 (91·8%)	4 (8·2%)	0·26 (0·10-0·66) P=0·005	0·28 (0·11-0·70) P=0·007
31-50 N=425	290 (68·2%)	135 (31·8%)	Ref -	Ref -
51-70 N=707	467 (66·1%)	240 (34·0%)	1·07 (0·90-1·27) P=0·45	1·06 (0·90-1·26) P=0·48
>70 N=141	72 (51·1%)	69 (48·9%)	1·54 (1·24-1·92) P<0·001	1·44 (1·16-1·79) P=0·001
Male – n (%) N=956	625 (65·4%)	331 (34·6%)	1·08 (0·91-1·29) P=0·37	1·08 (0·92-1·28) P=0·35
BMI – n (%)**				
<18·5 N=232	140 (60·3%)	92 (39·7%)	1·22 (1·01-1·47) P=0·04	1·16 (0·97-1·40) P=0·10
18·5-24·9 N=779	525 (67·4%)	254 (32·6%)	Ref -	Ref -
25·0-29·9 N=189	136 (72·0%)	53 (28·0%)	0·86 (0·67-1·10) P=0·24	0·90 (0·70-1·14) P=0·37
≥30·0 N=45	32 (71·1%)	13 (28·9%)	0·89 (0·55-1·42) P=0·61	0·91 (0·60-1·39) P=0·67
Referred from other facility – n (%) N=827	512 (61·9%)	315 (38·1%)	1·42 (1·20-1·68) P<0·001	1·33 (1·12-1·58) P=0·001
**Underlying conditions**				
Diabetes – n (%) N=927	635 (68·5%)	292 (31·5%)	0·80 (0·68-0·93) P=0·004	0·60 (0·51-0·71) P<0·001
Hypertension – n (%) N=423	257 (60·8%)	166 (39·2%)	1·25 (1·07-1·46) P=0·004	1·07 (0·91-1·26) P=0·43
Chronic kidney disease and renal calculi – n (%) N=228	123 (54·0%)	105 (46·1%)	1·46 (1·24-1·73) P<0·001	1·20 (0·99-1·45) P=0·07
Dyslipidemia – n (%) N=97	58 (59·8%)	39 (40·2%)	1·20 (0·93-1·55) P=0·15	1·19 (0·93-1·53) P=0·16
Chronic lung disease – n (%) N=164	92 (56·1%)	72 (43·9%)	1·35 (1·12-1·64) P=0·002	1·01 (0·83-1·24) P=0·91
Gout – n (%) N=58	25 (43·1%)	33 (56·9%)	1·73 (1·37-2·20) P<0·001	1·48 (1·17-1·87) P=0·001
Liver disease – n (%) N=48	21 (43·8%)	27 (56·3%)	1·70 (1·31-2·21) P<0·001	1·33 (1·00-1·77) P=0·05
Heart disease – n (%) N=62	28 (45·2%)	34 (54·8%)	1·67 (1·31-2·12) P<0·001	1·15 (0·88-1·50) P=0·32

Stroke or vascular disease – n (%) N=36	12 (33·3%)	24 (66·7%)	2·02 (1·58-2·58) P<0·001	1·37 (1·04-1·81) P=0·03
Cancer – n (%) N=49	24 (49·0%)	25 (51·0%)	1·54 (1·15-2·04) P=0·003	1·42 (1·06-1·91) P=0·02
Alcohol use disorder – n (%) N=66	34 (51·5%)	32 (48·5%)	1·46 (1·13-1·90) P=0·004	1·19 (0·89-1·58) P=0·23
Thalassemia – n (%) N=35	28 (80·0%)	7 (20·0%)	0·58 (0·30-1·14) P=0·11	0·74 (0·37-1·47) P=0·39
Autoimmune disease – n (%) N=26	19 (73·1%)	7 (26·9%)	0·79 (·42-1·50) P=0·47	0·94 (0·48-1·85) P=0·86
HIV – n (%) N=23	13 (56·5%)	10 (43·5%)	1·29 (0·80-2·07) P=0·29	1·36 (0·83-2·22) P=0·22
Anaemia – n (%) N=18	13 (72·2%)	5 (27·8%)	0·82 (0·39-1·73) P=0·60	0·90 (0·41-1·95) P=0·79
Chronic corticosteroid therapy – n (%) N=50	27 (54·0%)	23 (46·0%)	1·38 (1·01-1·88) P=0·04	1·49 (1·10-2·02) P=0·01
Previous melioidosis – n (%) N=63	35 (55·6%)	28 (44·4%)	1·33 (1·00-1·78) P=0·05	1·37 (1·05-1·79) P=0·02
No melioidosis risk factors – n (%) N=171	119 (69·6%)	52 (30·4%)	0·88 (0·69-1·12) P=0·31	0·93 (0·73-1·19) P=0·56
**Clinical Presentations**				
Lung infection – n (%) N=553	300 (54·3%)	253 (45·8%)	1·80 (1·55-2·10) P<0·001	1·61 (1·39-1·88) P<0·001
Skin/soft tissue infection – n (%) N=299	242 (80·9%)	57 (19·1%)	0·50 (0·39-0·64) P<0·001	0·56 (0·44-0·72) P<0·001
Bacteremia without focus – n (%) N=268	168 (62·7%)	100 (37·3%)	1·13 (0·9-1·35) P=0·18	1·11 (0·93-1·32) P=0·26
Intra-abdominal infection – n (%) N=209	162 (77·5%)	47 (22·5%)	0·62 (0·48-0·81) P<0·001	0·68 (0·52-0·89) P=0·005
Genitourinary tract infection – n (%) N=170	98 (57·7%)	72 (42·4%)	1·30 (1·07-1·58) P=0·009	1·32 (1·09-1·59) P=0·005
Septic arthritis – n (%) N=105	82 (78·1%)	23 (21·9%)	0·63 (0·43-0·91) P=0·01	0·65 (0·45-0·94) P=0·02
Osteomyelitis – n (%) N=11	9 (81·8%)	2 (18·2%)	0·53 (0·15-1·88) P=0·33	0·64 (0·20-2·07) P=0·46
Neurological infection – n (%) N=11	8 (72·7%)	3 (27·3%)	0·80 (0·31-2·12) P=0·66	0·93 (0·38-2·29) P=0·87
**Positive culture for *B. pseudomallei***				
Blood – n (%) N=1021	633 (62·0%)	388 (38·0%)	1·91 (1·50-2·42) P<0·001	1·79 (1·41-2·26) P<0·001
Respiratory tract sample – n (%) N=252	130 (51·6%)	122 (48·4%)	1·59 (1·36-1·86) P<0·001	1·42 (1·21-1·66) P<0·001
Urine – n (%) N=66	32 (48·5%)	34 (51·5%)	1·56 (1·22-2·00) P<0·001	1·39 (1·08-1·79) P=0·01

**Clinical Management**				
Received IV antibiotic active against *B. pseudomallei* – n (%)<br>N=1277	847 (66.3%)	430 (33·7%)	0·84 (0·58-1·21%)<br>P=0·36	0·90 (0·63-1·29)<br>P=0·57
Days to receipt of IV antibiotic active against *B. pseudomallei* – median (IQR)	0 (0-2)	0 (0-2)	0·99 (0·96-1·02)<br>P=0·56	0·99 (0·96-1·03)<br>P=0·76
Received vasopressor/inotrope<br>N=438	155 (35·4%)	283 (64·6%)	3·46 (2·97-4·04)<br>P<0·001	3·43 (2·94-4·00)<br>P<0·001
Received mechanical ventilation<br>N=543	216 (39·8%)	327 (60·2%)	3·88 (3·25-4·63)<br>P<0·001	3·74 (3·12-4·50)<br>P<0·001
ICU admission – n (%)<br>N=377	158 (41·9%)	219 (58·1%)	2·40 (2·08-2·76)<br>P<0·001	2·41 (2·08-2·79)<br>P<0·001

RR, relative risk; IQR, interquartile range; BMI, body mass index; IV, intravenous; ICU, Intensive Care Unit. Vital status at one month was not known for 30 of 1352 patients, BMI data is missing for 77 individuals, and 1277 patients received an IV antibiotic active against *B. pseudomallei* so total n=1322 for all exposures except BMI (n=1245) and days to receipt of IV antibiotic active against *B. pseudomallei* (n=1277). In total, 874 (66·1%) patients survived and 448 (33·9%) patients died at one year.

* 31-50 years is the reference group.

** 18·5-24·9 is the reference group; BMI data is missing for 79 individuals.

*** Adjusted models include age, sex, referral from other facility, time to enrollment, combined comorbidity index (except for no melioidosis risk factors model), and site as covariates.

Table S9. Diabetic therapies and relative risk of death at one month and at one year

Therapy	1 month mortality Unadjusted RR (95% CI) P value	1 month mortality Adjusted RR** (95% CI) P value	1 year mortality Unadjusted RR (95% CI) P value	1 year mortality Adjusted RR** (95% CI) P value
**Relative risk of death for individual oral medications among diabetic patients**				
Glipizide N=236 (24·8%)	0·98 (0·75-1·28) P=0·86	0·97 (0·74-1·26) P=0·79	0·92 (0·74-1·16) P=0·49	0·88 (0·70-1·09) P=0·24
Glyburide N=33 (3·5%)	0·38 (0·13-1·13) P=0·08	0·41 (0·15-1·10) P=0·08	0·57 (0·27-1·18) P=0·13	0·59 (0·30-1·18) P=0·14
Any sulfonylurea N=267 (28·1%)	0·87 (0·66-1·13) P=0·29	0·87 (0·67-1·13) P=0·29	0·85 (0·69-1·07) P=0·16	0·82 (0·66-1·02) P=0·07
Metformin N=267 (28·1%)	1·01 (0·78-1·31) P=0·94	0·99 (0·78-1·27) P=0·97	0·94 (0·76-1·16) P=0·55	0·92 (0·75-1·14) P=0·47
Sulfonylurea plus unknown diabetes drug N=391 (41·1%)	0·89 (0·70-1·13) P=0·34	0·86 (0·68-1·10) P=0·23	1·00 (0·83-1·21) P=1·00	0·94 (0·77-1·14) P=0·52
Metformin plus unknown diabetes drug N=391 (41·1%)	1·01 (0·80-1·28) P=0·93	0·97 (0·76-1·22) P=0·78	1·08 (0·89-1·30) P=0·45	1·04 (0·85-1·26) P=0·70
**Risk of death for diabetic patients taking increasingly intensive therapy relative to non-diabetic patients***				
No diabetes N=401	Ref -	Ref -	Ref -	Ref -
Diabetes - no drugs N=321	0·75 (0·58-0·97) P=0·03	0·57 (0·43-0·75) P<0·001	0·71 (0·57-0·88) P=0·002	0·58 (0·46-0·73) P<0·001
Diabetes - Any one oral drug N=241	0·73 (0·55-0·98) P=0·04	0·51 (0·37-0·70) P<0·001	0·82 (0·66-1·03) P=0·08	0·59 (0·45-0·75) P<0·001
Diabetes - Two oral drugs N=144	0·65 (0·45-0·95) P=0·03	0·48 (0·33-0·70) P<0·001	0·62 (0·45-0·84) P=0·002	0·48 (0·35-0·65) P<0·001
Diabetes - Insulin and none or any oral drug N=245	1·07 (0·84-1·36) P=0·60	0·70 (0·53-0·94) P=0·02	1·00 (0·82-1·22) P=0·99	0·70 (0·55-0·88) P=0·002

RR, relative risk. Percents are calculated for 951 diabetics. 124 patients were taking an unknown diabetic drug that was binned with either sulfonylurea or metformin in sensitivity analyses. 24 patients were taking pioglitazone; this was not considered in these analyses.

* Increasingly intensive therapy (ranging from diet control/no drugs to insulin) is a proxy for severity of diabetes. No diabetes is the reference group. One month vital status was not known for 7 of 1352 patients; 1010 (75·1%) patients survived and 335 (24·9%) patients died. One year vital status was not known for 30 of 1352 patients; 874 (66·1%) survived and 448 (33·9%) died.

** Adjusted models include age, sex, BMI, hypertension, dyslipidemia, chronic kidney disease, heart disease, stroke or vascular disease, combined comorbidity index, referral from other facility, time to enrollment, and site as covariates.

Table S10. Characteristics of melioidosis patients with or without diabetes

Patient characteristics	No Diabetes (n = 401)	Diabetes (n = 951)	P value
Age in years, grouped – n (%)			
15-30	28 (7·0%)	22 (2·3%)	<0·001
31-50	108 (26·9%)	328 (34·5%)	
51-70	201 (50·1%)	523 (55·0%)	
>70	64 (16·0%)	78 (8·2%)	
Male – n (%)	311 (77·6%)	664 (69·8%)	0·004
BMI, grouped – n (%)*			
<18·5	95 (25·1%)	142 (15·9%)	<0·001
18·5-24·9	241 (63·8%)	556 (62·2%)	
25·0-29·9	36 (9·5%)	157 (17·6%)	
≥30·0	6 (1·6%)	39 (4·4%)	
**Underlying conditions**			
Hypertension – n (%)	70 (17·5%)	359 (37·8%)	<0·001
Chronic kidney disease and renal calculi – n (%)	75 (18·7%)	154 (16·2%)	0·26
Dyslipidemia – n (%)	10 (2·5%)	90 (9·5%)	<0·001
Chronic lung disease – n (%)	79 (19·7%)	85 (8·9%)	<0·001
Gout – n (%)	29 (7·2%)	29 (3·1%)	0·001
Liver disease – n (%)	16 (4·0%)	32 (3·4%)	0·57
Heart disease – n (%)	22 (5·4%)	40 (4·2%)	0·30
Stroke or vascular disease – n (%)	11 (2·7%)	29 (3·1%)	0·76
Cancer – n (%)	30 (7·5%)	19 (2·0%)	<0·001
Alcohol use disorder – n (%)	27 (6·7%)	40 (4·2%)	0·05
Thalassemia – n (%)	21 (5·2%)	15 (1·6%)	<0·001
Autoimmune disease – n (%)	17 (4·2%)	9 (1·0%)	<0·001
HIV – n (%)	14 (3·5%)	10 (1·1%)	0·002
Anemia – n (%)	9 (2·2%)	9 (1·0%)	0·07
Chronic corticosteroid therapy – n (%)	23 (5·7%)	28 (2·9%)	0·01

* BMI data are missing for 80 individuals.

Table S11. Relative risk of death within one year after enrollment among patients with diabetes or other melioidosis risk factors

Patient characteristics	Survived	Died	Unadjusted RR (95% CI) P value	Adjusted RR* (95% CI) P value
No risk factors<br>N=171	119 (69·6%)	52 (30·4%)	Ref<br>-	Ref<br>-
Risk factors other than diabetes<br>N=224	120 (53·6%)	104 (46·4%)	1·53 (1·17-1·99)<br>P=0·002	1·45 (1·10-1·88)<br>P=0·007
Diabetes<br>N=927	635 (68·5%)	292 (31·5%)	1·04 (0·81-1·32)<br>P=0·78	0·97 (0·76-1·25)<br>P=0·84

Non-diabetes risk factors for acquisition of melioidosis are alcohol use disorder, chronic lung disease, chronic kidney disease, renal calculi, heart disease, thalassaemia, cancer, and chronic corticosteroid therapy. Of 1322 patients, 874 (66·1%) survived and 448 (33·9%) died at one year.
* Adjusted model includes age, sex, referral from other facility, time to enrollment, and site as covariates.

Table S12. Relative risk of post-discharge death within one year after enrollment among patients with specific clinical presentations

Clinical Presentations	Survivors	Non-survivors	Unadjusted RR (95% CI) P value	Adjusted RR* (95% CI) P value
Lung infection – n (%) N=312	278 (89·1%)	34 (10·9%)	1·20 (0·80-1·81) P=0·37	1·15 (0·77-1·72) P=0·50
Skin/soft tissue infection – n (%) N=240	227 (94·6%)	13 (5·4%)	0·48 (0·27-0·85) P=0·01	0·58 (0·33-1·01) P=0·06
Bacteremia without focus – n (%) N=180	160 (88·9%)	20 (11·1%)	1·19 (0·74-1·91) P=0·47	1·04 (0·64-1·69) P=0·86

RR, relative risk; only the most common clinical presentations were analysed. Patients discharged alive and who were alive at one month were included. Total n=909 for all exposures; 821 (90·3%) patients survived and 88 (9·7%) patients died at one year.
* Adjusted models include age, sex, diabetes, heart disease, chronic kidney disease and renal calculi, cancer, gout, chronic corticosteroid use, combined comorbidity index, length of stay, time to enrollment, and site as covariates.

Table S13. Relative risk of post-discharge death within one year after enrollment in patients taking TMP-SMX eradication therapy

Follow up time point	Survived	Died	Unadjusted RR (95% CI) P value	Adjusted RR* (95% CI) P value
Taking TMP-SMX at one month N=781/877	721 (92·3%)	60 (7·7%)	0·31 (0·20-0·47) P<0·001	0·32 (0·21-0·51) P<0·001
Taking TMP-SMX at two months N=773/887	724 (93·7%)	49 (6·3%)	0·52 (0·29-0·90) P=0·02	0·52 (0·26-1.02) P=0·06
Taking TMP-SMX at four months N=727/849	703 (96·7%)	24 (3·3%)	0·37 (0·18-0·73) P=0·004	0·38 (0·18-0·80) P=0·01

RR, relative risk. Denominators are patients for whom one year vital status was known and who were discharged from hospital alive, and who were contacted at each follow up time point. Of 877 patients contacted at one month, 793 survived and 84 died at one year. Of 887 patients contacted at two months, 824 survived and 63 died at one year. Of 849 patients contacted at four months, 814 survived and 35 died at one year.
* Adjusted models include age, sex, diabetes, heart disease, kidney disease and renal calculi, cancer, gout, chronic corticosteroid use, combined comorbidity index, length of stay, time to enrollment, and site as covariates.

Figure Legends

Figure S1. Diagnostic and therapeutic drainage procedures performed. The number of patients undergoing a procedure is reported. Patients undergoing the same procedure more than once are only counted once. GU, genitourinary; EGD, esophagogastroduodenoscopy.

Figure S2. Time to readmission after discharge. Histogram of days to readmission for all readmitted patients.

Figure S3. Symptoms reported following hospital discharge. The percent of patients contacted at each follow up time point reporting specific symptoms.

Figure S4. One year survival by melioidosis risk factors. DM: diabetes. Non-DM: melioidosis risk factors other than diabetes. None: no melioidosis risk factors. Non-diabetes risk factors were alcohol use disorder, chronic lung disease, chronic kidney disease, heart disease, thalassaemia, cancer, and chronic corticosteroid therapy. P<0·001 by the logrank test.

Figure S5. Cumulative incidence of hospital readmission by melioidosis risk factors. DM: diabetes. Non-DM: melioidosis risk factors other than diabetes. None: no melioidosis risk factors. Non-diabetes risk factors were alcohol use disorder, chronic lung disease, chronic kidney disease, heart disease, thalassaemia, cancer, and chronic corticosteroid therapy. Competing risk analysis considering death as the competing risk. Subhazard ratio for readmission for non-diabetes risk factors relative to no risk factors is 2·85 (95% CI: 1·77-4·56). Subhazard ratio for readmission for diabetes relative to no risk factors is 1·31 (95% CI: 0·85-2·01).

Figure S1.

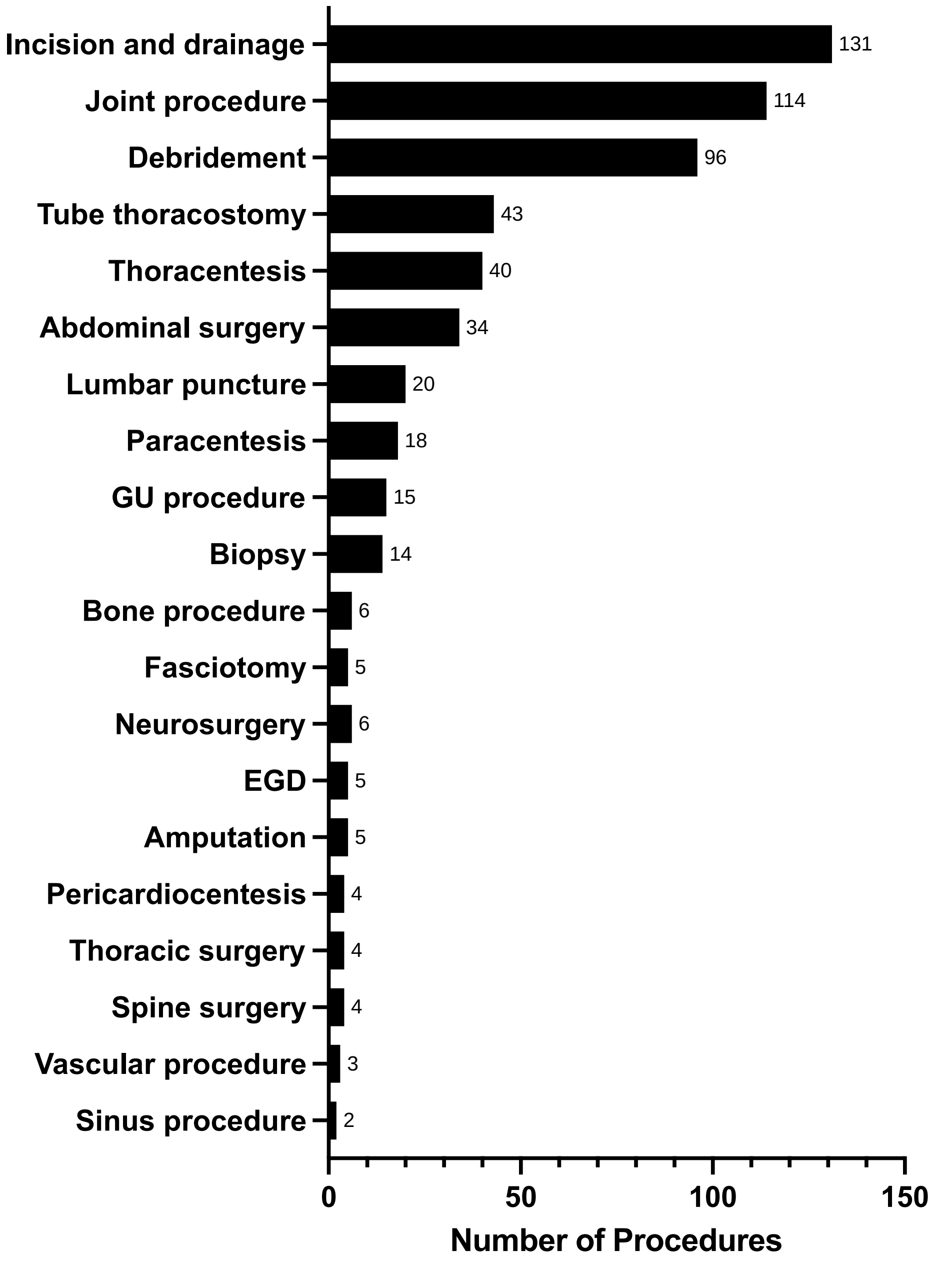
Incision and drainage
Joint procedure
Debridement
Tube thoracostomy
Thoracentesis
Abdominal surgery
Lumbar puncture
Paracentesis
GU procedure
Biopsy
Bone procedure
Fasciotomy
Neurosurgery
EGD
Amputation
Pericardiocentesis
Thoracic surgery
Spine surgery
Vascular procedure
Sinus procedure
131
114
96
43
40
34
20
18
15
14
6
5
6
5
5
4
4
4
3
2
0
50
100
150
Number of Procedures

Figure S2.

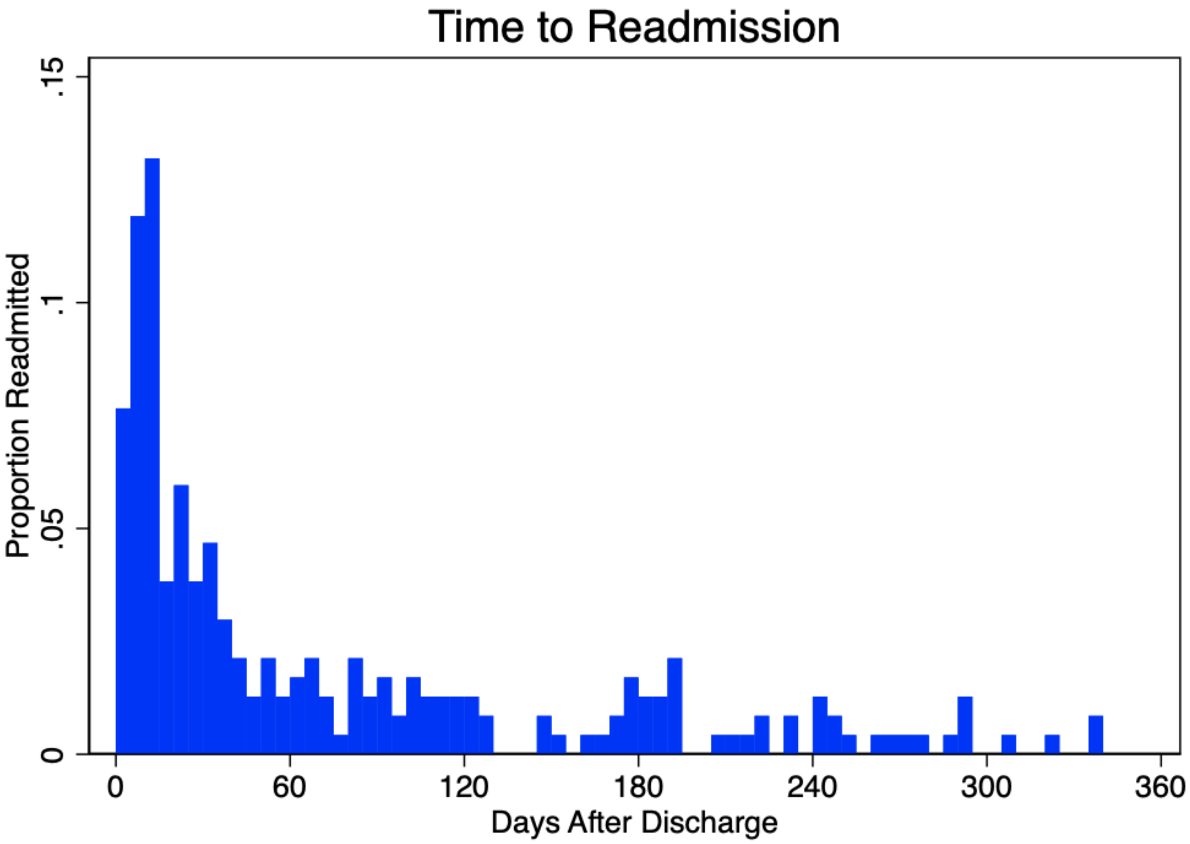

Time to Readmission
Proportion Readmitted
0
.05
.1
.15
0
60
120
180
240
300
360
Days After Discharge

Figure S3.

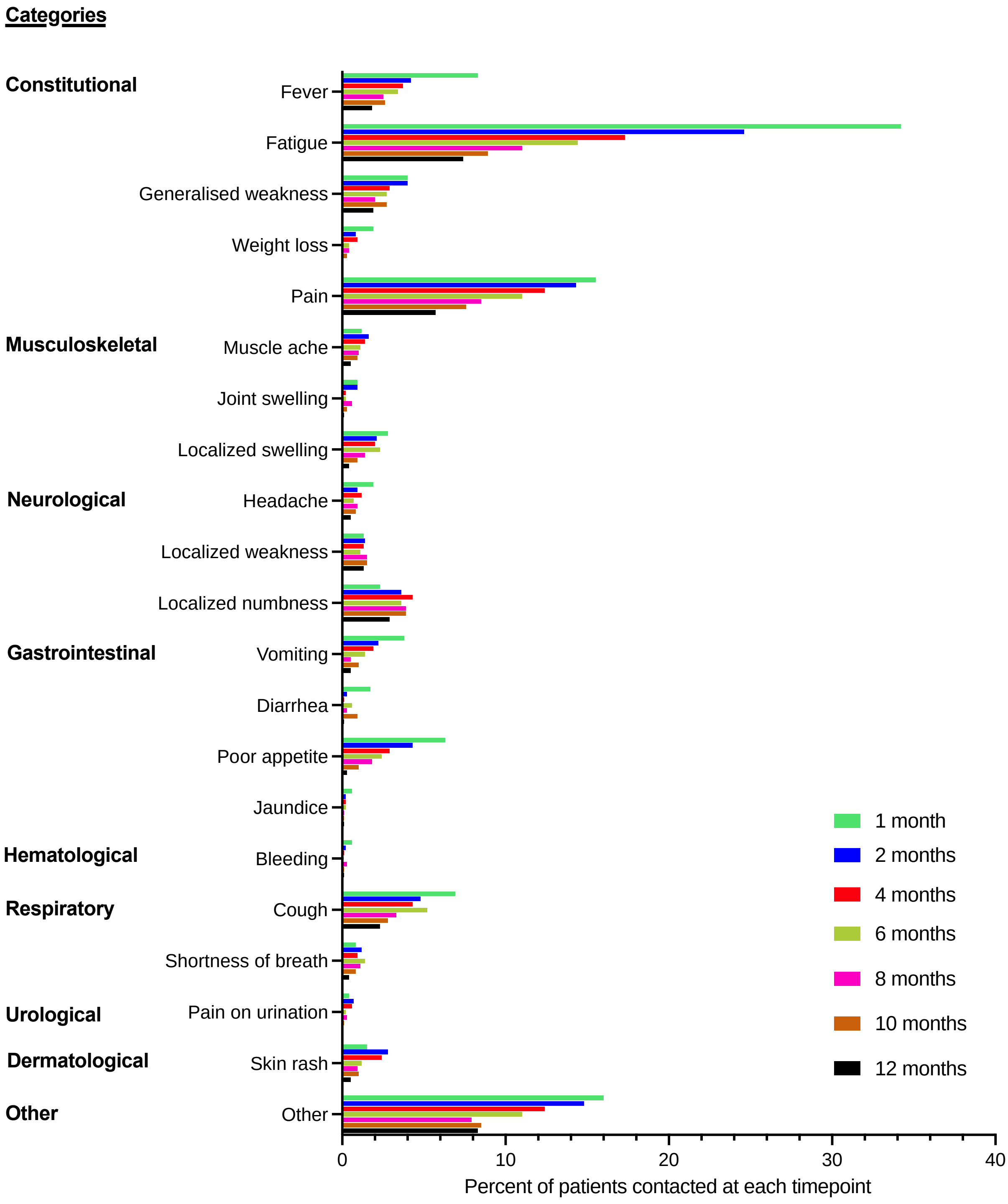
Categories
Constitutional
Fever
Fatigue
Generalised weakness
Weight loss
Pain
Musculoskeletal
Muscle ache
Joint swelling
Localized swelling
Neurological
Headache
Localized weakness
Localized numbness
Gastrointestinal
Vomiting
Diarrhea
Poor appetite
Jaundice
Hematological
Bleeding
Respiratory
Cough
Shortness of breath
Urological
Pain on urination
Dermatological
Skin rash
Other
Other
0
10
20
30
40
Percent of patients contacted at each timepoint
1 month
2 months
4 months
6 months
8 months
10 months
12 months

Figure S4.

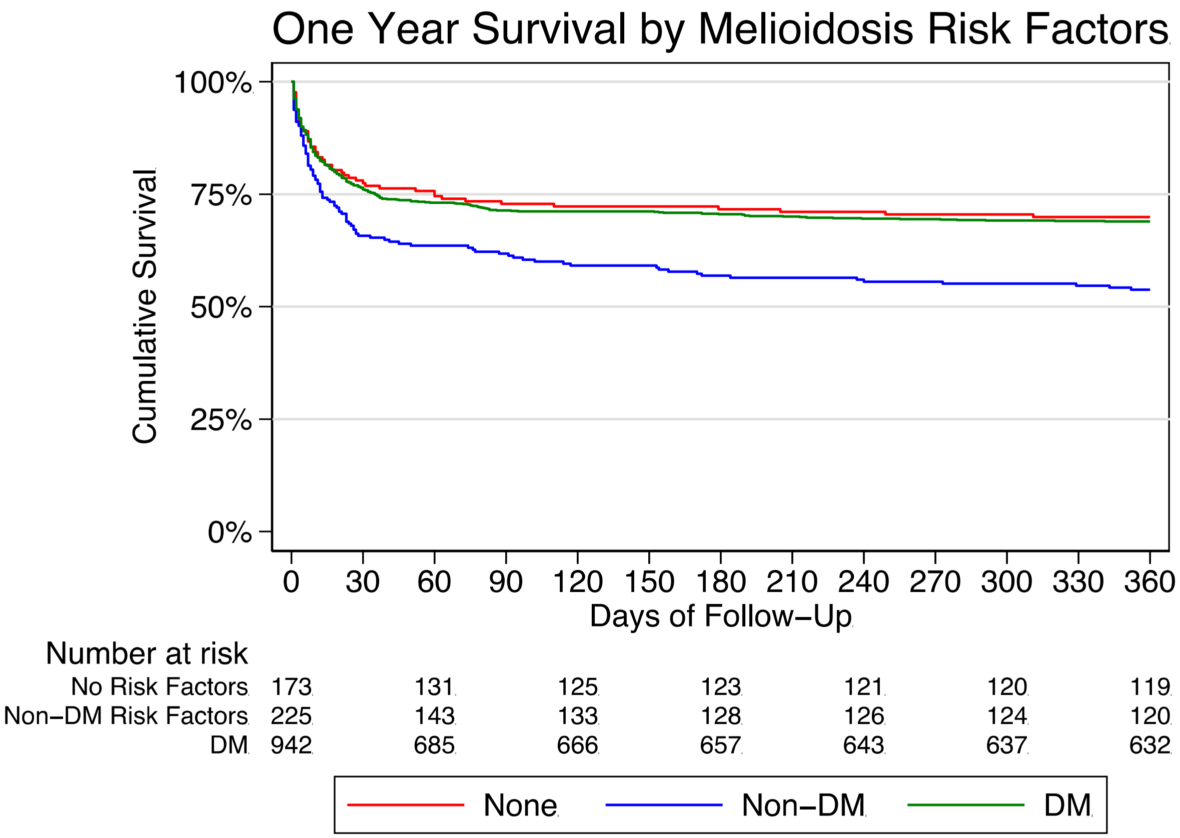
One Year Survival by Melioidosis Risk Factors
Cumulative Survival
100%
75%
50%
25%
0%
0 30 60 90 120 150 180 210 240 270 300 330 360
Days of Follow−Up
Number at risk
No Risk Factors 173 131 125 123 121 120 119
Non−DM Risk Factors 225 143 133 128 126 124 120
DM 942 685 666 657 643 637 632
None
Non−DM
DM

Figure S5.

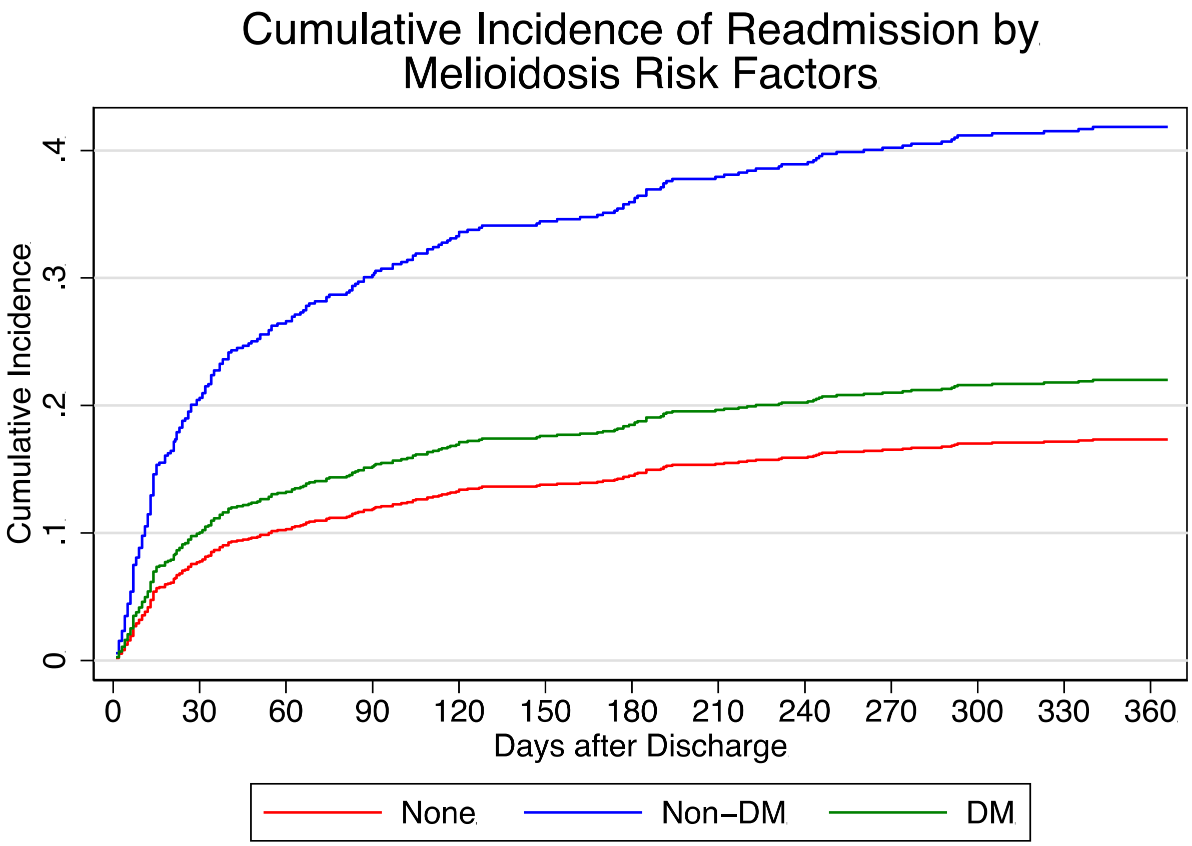

Cumulative Incidence of Readmission by
Melioidosis Risk Factors
Cumulative Incidence
0
.1
.2
.3
.4
0
30
60
90
120
150
180
210
240
270
300
330
360
Days after Discharge
None
Non−DM
DM

Data Sharing

Following publication, summary data, case report forms, and consent forms from this study are available upon request from the authors.
